# Reservoir computing on a silicon platform with a ferroelectric field-effect transistor

**DOI:** 10.1038/s44172-022-00021-8

**Published:** 2022-08-05

**Authors:** Kasidit Toprasertpong, Eishin Nako, Zeyu Wang, Ryosho Nakane, Mitsuru Takenaka, Shinichi Takagi

**Affiliations:** Department of Electrical Engineering and Information Systems, The University of Tokyo, 7-3-1 Hongo, Bunkyo-ku, Tokyo, 113-8656 Japan

**Keywords:** Electrical and electronic engineering, Electronics, photonics and device physics

## Abstract

Reservoir computing offers efficient processing of time-series data with exceptionally low training cost for real-time computing in edge devices where energy and hardware resources are limited. Here, we report reservoir computing hardware based on a ferroelectric field-effect transistor (FeFET) consisting of silicon and ferroelectric hafnium zirconium oxide. The rich dynamics originating from the ferroelectric polarization dynamics and polarization-charge coupling are the keys leading to the essential properties for reservoir computing: the short-term memory and high-dimensional nonlinear transform function. We demonstrate that an FeFET-based reservoir computing system can successfully solve computational tasks on time-series data processing including nonlinear time series prediction after training with simple regression. Due to the FeFET’s high feasibility of implementation on the silicon platform, the systems have flexibility in both device- and circuit-level designs, and have a high potential for on-chip integration with existing computing technologies towards the realization of advanced intelligent systems.

## Abstract (web summary)

Kasidit Toprasertpong and colleagues describe reservoir computing hardware with potential for on-chip integration with existing computing technologies. The approach is based on a ferroelectric field-effect transistor, and can solve computational tasks on time series data including nonlinear time series prediction after training with simple regression.

## Introduction

Rapid increasing demand for edge computing, particularly edge artificial intelligence (AI), requires hardware that can efficiently process huge volume of data acquired at edge devices with low computational cost and energy consumption. The scaling limitation of the complementary metal-oxide-semiconductor (CMOS) technology and the inefficient AI computing in the conventional von Neumann architecture emphasize the need of novel computing technologies for AI processing, for instance, with the use of emerging electron devices in computing-in-memory architectures^1,2^. It is particularly challenging for the implementation of edge-AI computing technologies that can be efficiently trained and used to process time-series data, which are one main type of data acquired from sensors of edge devices.

Reservoir computing is a computational framework that can process time-series data with extremely-low computational cost. A reservoir computing system is composed of a reservoir part containing history-dependent and nonlinear dynamics, and a readout part consisting of an output layer and direct weighted connections with the reservoir states^3,4^. The fact that there is only one layer of adjustable weights allows the training step to be carried out with simple models such as linear or ridge regressions (without computationally-heavy backpropagation), which is considerably promising for efficient online training^5^. A representative model of software-based reservoir computing is echo state networks (see Supplementary Fig. S1.1a), in which the nonlinear dynamics of the reservoir part are realized by recurrent neural networks with fixed-weight connections. However, the hardware implementation of echo state networks is not easy to achieve because of the large size and complicated connections in neural networks.

After years from the first proposal of reservoir computing, hardware-oriented reservoir computing, called physical reservoir computing, has emerged, in which the reservoir part is implemented by a physical system (called physical reservoir; see Supplementary Fig. S1.1b). The most remarkable feature is that the physical reservoir is simply required to have a dynamical function that is driven by the input as well as the recent input history, and it can nonlinearly transform time-series inputs to spatiotemporal node states with a higher dimension^6^; therefore, the hardware implementation becomes feasible. These properties are usually referred to as short-term memory and nonlinearity, respectively. So far, physical reservoir computing has been studied using various physical reservoirs, including soft materials^7^, molecular networks^8^, electrochemical devices^9^, atomic switching networks^10^, photonic systems^11–13^, spin torque oscillators^14,15^, spin waves^16,17^, and memristors^18,19^. A number of demonstrations of practical applications such as time-series prediction^7,8,11,12,18,19^, early illness detection^9^, and spoken digit recognition^11–14,19^ suggests that physical reservoir computing has a high potential as a disruptive hardware technology in next-generation edge-AI computing where the power and memory-capacity resources are restricted. On the other hand, despite several demonstrations, there is still a lack of understanding of the required properties for physical reservoirs, so physical reservoirs whose properties are easily adjustable depending on target tasks are promising for establishing the design guideline of physical reservoirs. Furthermore, for practical applications of edge-AI computing, a physical reservoir based on materials and/or devices compatible with the silicon CMOS technology, such as memristors^18,19^ and ferroelectric devices^20,21^, is strongly required for on-chip integration together with other memory and computing systems to accomplish functions required by edge-AI.

In this article, we report reservoir computing hardware based on a CMOS-compatible ferroelectric field-effect transistor (FeFET). An FeFET is one type of a field-effect transistor where a ferroelectric material is used as a gate insulator. Among available ferroelectric materials, hafnium oxide (HfO_2_)-based ferroelectric materials are known to be highly compatible materials in the silicon CMOS platform^22^ and can be easily implemented as gate insulators of FeFETs^23^. Large arrays integration of FeFETs in the same platform as logic CMOS have been successfully demonstrated^24^. HfO_2_-based FeFETs have been investigated in a wide range of applications including logic^25^, nonvolatile memory array^24^, ternary content-addressable memory^26^, and computing-in-memory^27–29^, most of which rely on the static properties of the ferroelectric polarization. The use of FeFETs for reservoir computing extracts the unexplored potential of their rich dynamics including the domain dynamics and the coupling dynamics between polarizations and charges for AI computing, opening up an alternative application of FeFETs. Furthermore, the characteristics of FeFET-based physical reservoirs can be sensitively controlled by a wide range of design flexibility from the material level, device structures, and the applicability to a circuit-level implementation by multiple-device integration. Compared with 2-terminal memristors typically employed to implement CMOS-compatible reservoir computing, FeFETs are fundamentally multi-terminal devices, allowing us to exploit both the temporal and spatial dynamics of polarization and charges for reservoir computing, and notably offer a larger design space to tune the reservoir properties through their multi-terminal structure. It is worth noting that these features provide not only promising AI hardware, but also contributes greatly to deep understanding and design guideline of physical reservoirs required for the fundamental research and applications of physical reservoir computing.

## Results

### FeFET as a physical reservoir device

**Fig. 1:**
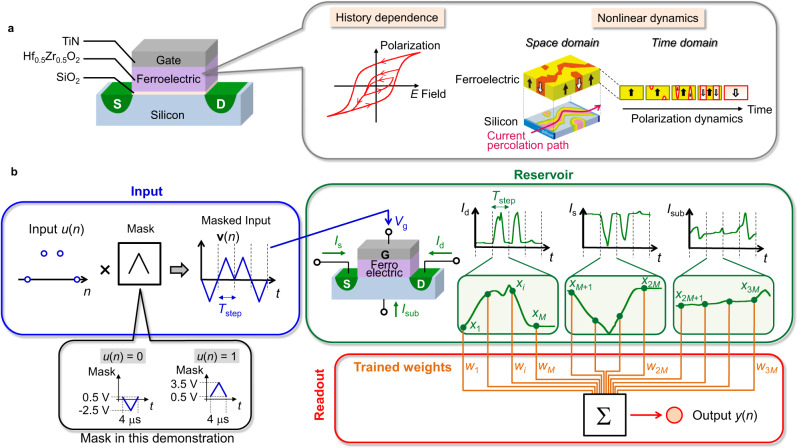
Reservoir computing using an FeFET. **a** Schematic of an FeFET with a ferroelectric gate insulator. One possible material combination of a CMOS-compatible FeFET employed in this work is also shown. G, S, and D indicate the gate metal, source region, and drain region, respectively. The yellow and red regions in the ferroelectric layer depict the up-polarized ferroelectric domain and the down-polarized ferroelectric domain, respectively. The ferroelectric layer possesses the hysteresic property and the nonlinear dynamics (see Supplementary Section 3–4 for more details). **b** FeFET-based reservoir computing system and operating scheme. The input time-series signal *u*(*n*) with a time step of *T*_step_ (= 4 μs in this demonstration) is transformed using a temporal mask function into a voltage waveform **v**(*n*), and is subsequently applied to the gate of the FeFET. The mask function in this demonstration, shown in the lower-left corner, is a triangular voltage waveform with an amplitude of 3 V and an offset of 0.5 V. The drain current *I*_d_(*t*), source current *I*_s_(*t*) and substrate current *I*_sub_(*t*) are sampled by *M* points (=200 in this demonstration) each to construct a set of 3 *M* virtual nodes **x**(*n*) = [*x*_1_(*n*), …, *x*_*i*_(*n*), …, *x*_3*M*_(*n*)] of the reservoir. The system output *y*(*n*) is obtained by a weighted sum of the reservoir nodes with the trained weights.

A typical structure of a HfO_2_-based FeFET is shown in Fig. 1a. Among many possible material combinations for an FeFET, we employed TiN as a gate metal, 10-nm-thick hafnium zirconium oxide (Hf_0.5_Zr_0.5_O_2_) as a ferroelectric insulator, chemical oxide as a high-quality interfacial layer^30^, and silicon as a semiconductor channel material for demonstration in this work. A transmission electron microscope (TEM) image, the *I*_d_-*V*_g_ characteristics, and the polarization characteristics^31^ are shown in Supplementary Figs. S2.1–S2.3. The electrostatic of the semiconductor (silicon) channel and consequently the flowing current are controlled by both the gate voltage *V*_g_ and the polarization state *P* of the ferroelectric insulator, while the polarization state *P* is known to be strongly dependent on the history of electric field *E* applied on the ferroelectric insulator, and hence the history of input *V*_g_. (See Supplementary Fig. S3.1). In this way, the flowing current can be expressed in terms of both the present input (*V*_g_) and the input history-dependent internal state (*P*). Furthermore, nonlinear polarization dynamics and polarization/charge interaction^32,33^ in an FeFET lead to the nonlinearity of the flowing current. Even though the operation mechanism of a HfO_2_-based FeFET is not yet fully understood and is still under intensive investigation by researchers, there are a number of reports on a variety of polarization dynamics in HfO_2_-based ferroelectric thin films. It has been reported that the polarization domain nucleation depending on the accumulated input^34^ and the nonlinear domain growth through domain wall motion^35,36^ in the HfO_2_-based ferroelectric insulator are key transient mechanisms under electric field (See Supplementary Fig. S4.1). The interaction between ferroelectric domains at the vicinity of domain walls would also increase the complexity of the domain growth and the polarization reversal mechanisms^35^, which is expected to promote the nonlinearity of polarization dynamics in both the space and time domains. Furthermore, the time scale of the polarization reversal process is known to have a nonlinear dependency on the applied voltage magnitude^37^. As a result, the current flowing from each terminal of a multi-terminal FeFET exhibits the temporal nonlinear dynamics and the spatial distribution through the spatial interaction of polarization and the percolation path formed by the polarization state. In this way, we can say that the current responses of an FeFET to the input gate voltage *V*_g_ contain history-dependent and nonlinear dynamics, which can be used as reservoir states in the FeFET reservoir.

**Fig. 2:**
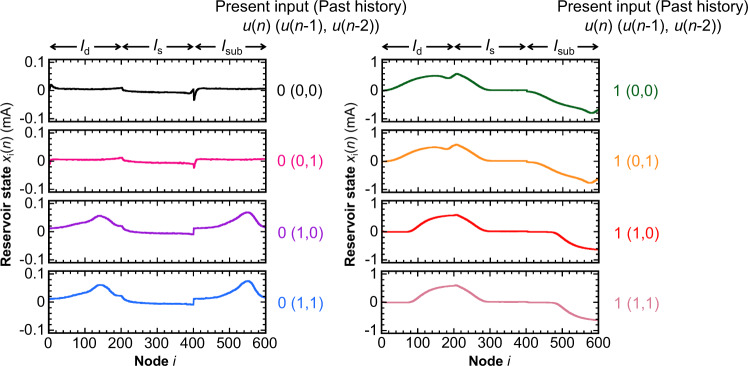
Internal states of FeFET reservoir. Reservoir-state components *x*_*i*_(*n*) are displayed for different present inputs *u*(*n*) and input histories *u*(*n*−1), *u*(*n*−2). The node *i* when 1 ≤ *i* ≤ 200 corresponds to the drain current *I*_d_, 201 ≤ *i* ≤ 400 corresponds to the source current *I*_s_, and 401 ≤ *i* ≤ 600 corresponds to the substrate current *I*_sub_.

Figure 1b shows the schematic of our reservoir computing system using an FeFET. (see Method for further details). A time-series input *u*(*n*) (discrete time step *n* = 1, 2, …) is converted into a voltage waveform **v**(*n*) through a predetermined function (masking procedure), which is subsequently applied to the gate of the FeFET to feed the input into the FeFET reservoir^20^. The reservoir states are readout through the measured current from the drain, source, and substrate contacts of the FeFET. For each contact, we employ the virtual node technique, which extracts sub-time-step data as additional nodes^38^. That is, the drain current *I*_d_(*t*), source current *I*_s_(*t*), and substrate current *I*_sub_(*t*) (*t*: continuous time) are sampled by *M* points (= 200 points in this demonstration) each per one-time step *n* to form a 3*M*-element reservoir-state vector **x**(*n*) as shown in Figs. 1b and 2. The reservoir computing system output *y*(*n*) (or *q*-element vector **y**(*n*) for *q*-output task, where *q* is 1, 2, …. Here, *q* = 1 for each task in this demonstration) is determined by the matrix multiplication between **x**(*n*) and the 3 *M* × *q* weight matrix **W**. The weight matrix **W** is trained so that *y*(*n*) becomes as close as possible to the target value *d*(*n*) (or **d**(*n*)) of each given task. In contrast with the FeFET reservoir system in our previous report^20^ in which only *I*_d_(*t*) was used to form the reservoir state, the reservoir system proposed in this Article utilizes all three current terminals *I*_d_(*t*), *I*_s_(*t*), *I*_sub_(*t*) for reservoir computing. Since currents *I*_d_(*t*), *I*_s_(*t*), *I*_sub_(*t*) reflect different physical dynamics in the FeFET (see Supplementary Fig. S5.1 and ref. ^32^), utilizing these current components are expected to increase the dimensionality of the reservoir state needed for computing.

### Demonstration of high-dimensional nonlinear mapping

**Fig. 3:**
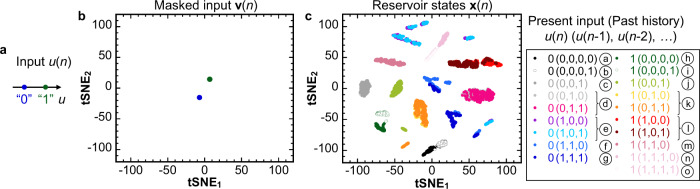
Visualization of high-dimensional transformation capability of an FeFET reservoir by t-SNE analysis. **a** Two patterns of binary input *u*(*n*). **b** t-SNE map of 4200 data points of masked input waveforms **v**(*n*), whose *j*th element *v*_*j*_(*n*) corresponds to the voltage waveform at time (*n* + *j*/*M*)*T*_step_ (*j* = 1, 2, …, *M*; *M* = 200). All the 4200 points in the plot gather into two groups, corresponding to the two patterns of masked input. **c** t-SNE map of 4200 data points of the reservoir states **x**(*n*) transformed from the inputs *u*(*n*). Each point is colored according to the value of the input *u*(*n*) and the recent input history *u*(*n*−1), *u*(*n*−2), *u*(*n*−3), and *u*(*n*−4). 15 groups of clusters can be observed as indicated by circled alphabets. The reservoir state **x**(*n*) of the FeFET reservoir is found to have a higher dimension than that of the time-series input *u*(*n*) owing to the high-dimensional nonlinear transformation capability of the FeFET reservoir.

The capability of an FeFET to nonlinearly transform the input into high-dimensional reservoir states is analyzed by visualizing the reservoir states using t-distributed stochastic neighbor embedding (t-SNE), which is a technique to reduce the data dimension while keeping the relation of distance among data to visualize a distribution of high-dimensional data^39^. Here, we carry out a t-SNE analysis when the input *u*(*n*) is binary, which is equivalent to two points in the *u* axis (Fig. 3a). Before discussing the reservoir states, we first examine the masked input waveform **v**(*n*) to be applied to the FeFET gate, which is converted from input signal *u*(*n*) in the same way as the data preprocess in Fig. 1b. The t-SNE plot of the masked input waveforms in Fig. 3b shows that all points gather into two groups similar to the scattering of *u*(*n*), confirming that the data preprocessing by a mask function does not increase the signal dimension since the same inputs *u*(*n*) are always converted into the same waveform **v**(*n*). (See Supplementary Fig. S6.1 for the effect of the measurement instrument on the data dimension).

On the other hand, it can be seen from Fig. 2 that the reservoir states **x**(*n*) are found to be different even when the present inputs *u*(*n*) are the same. (See Supplementary Fig. S7.1 for the comparison of **x**(*n*) with different *u*(*n*−2)). The t-SNE plot of the reservoir states **x**(*n*) in Fig. 3c shows that the reservoir states can be distinguished into a variety of different patterns, determined by the present input *u*(*n*), the earlier inputs *u*(*n*−1), *u*(*n*−2), and sometimes also *u*(*n*−3) and *u*(*n*−4). When the t-SNE map is labeled according to the input history, we can see that the reservoir states can be separated into approximately 15 groups, indicating that 15 patterns of reservoir states are distinguishable under binary input time-series. We employ the *k*-nearest neighbor (*k*NN) method to confirm the 15 distinguishable patterns of the reservoir states in a more quantitative manner. Classifying 600-element **x**(*n*) into the 15 patterns using *k*NN results in a classification error rate <0.05. This is evidence that the FeFET has the capability to nonlinearly transform the time-series input data (one dimension in this case) into the high-dimensional reservoir states, satisfying the required property of the reservoir part.

### Reservoir computing performance

**Fig. 4:**
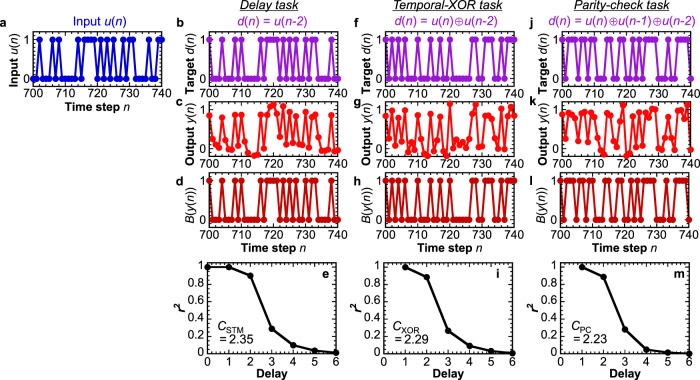
Reservoir computing results for delay, temporal-XOR, and parity-check tasks. **a** Input signal *u*(*n*) of the test data. Only 40 time steps are shown here. **b**–**d** Target output *d*(*n*) = *u*(*n*−2) (**b**), computed system output *y*(*n*) (**c**), and binarized output *B*(*y*(*n*)) (**d**) of the trained FeFET reservoir system for the 2-step delay task. Here, *B*(*y*(*n*)) = 0 when *y*(*n*) < 0.5 and *B*(*y*(*n*)) = 1 whe*n y*(*n*) ≥ 0.5. **e** Squared correlation coefficient *r*^2^ between the computed system output *y*(*n*) and the target output *d*(*n*) = *u*(*n*−*τ*) for the *τ*-step delay tasks. **f**–**h** Target signal *d*(*n*) = *u*(*n*) ⨁ *u*(*n*−2) **f**, computed system output *y*(*n*) (**g**) and binarized output *B*(*y*(*n*)) (**h**) for the two-step temporal-XOR task, where ⨁ is the XOR operation. **i** *r*^2^ between *y*(*n*) and *d*(*n*) = *u*(*n*) ⨁ *u*(*n*−*τ*) for the *τ*-step temporal-XOR task. **j**–**l** Target *d*(*n*) = *u*(*n*) ⨁ *u*(*n*−1) ⨁ *u*(*n*−2) (**j**)_,_ output *y*(*n*) (**k**) and binarized output *B*(*y*(*n*)) for the two-step parity-check task. **m** *r*^2^ between *y*(*n*) and *d*(*n*) = *u*(*n*) ⨁ *…* ⨁ *u*(*n*–*τ*) = ${\sum }_{l=0}^{\tau }u(n-l)({{{{\mathrm{mod}}}}}\,2)$ for the *τ*-step parity-check task. *C*_STM_, *C*_XOR_, and *C*_PC_, which are the sum of *r*^2^ for delay step *τ* ≥ 1, are shown in the corresponding figures.

We investigate the computational performance of the FeFET-based reservoir computing system when the time-series input *u*(*n*) is a random binary sequence as shown in Fig. 4a. For each given task, the weight matrix **W** is trained by the ridge regression using the task-specific target *d*(*n*), and the computing performance is evaluated by testing with an unseen dataset. The ridge parameter is optimized to mitigate the overfitting of **W** and maximize the reservoir computing performance (see Supplementary Figs. S8.1–S8.2 that the noisy feature of the matrix elements *W*_*i*_ disappears at the optimal ridge parameter). First, delay tasks were carried out to evaluate the short-term memory capacity (*C*_STM_), corresponding to the short-term memory characteristics^20,40^. In the delay tasks, the target output *d*(*n*) is the time-series of the input with a delay step *τ*, i.e., *d*(*n*) = *u*(*n*−*τ*) (Eq. (11)). After **W** is determined by training, the computational performance is evaluated using the squared correlation coefficient *r*^2^ between the reservoir computing system output *y*(*n*) and the target output *d*(*n*) when the test dataset is input (Fig. 4b, c for *τ* = 2). The results of *r*^2^ for different *τ* are summarized in Fig. 4e. It can be seen that the FeFET-based reservoir computing system can be trained to well reproduce the input *u*(*n*), the one-delay input *u*(*n*−1), and the two-delay input *u*(*n*−2). Although *r*^2^ at *τ* = 2 does not reach 1 due to a finite fluctuation in *y*(*n*) (see Fig. 4c), inserting a threshold of 0.5 (see Eq. (9)) as the final system output leads to an accuracy >98% (Fig. 4d). These results agree well with the t-SNE analysis in Fig. 3 that the reservoir states **x**(*n*) contain the information of *u*(*n*), *u*(*n*−1) and *u*(*n*−2), and thus the past information can be extracted by the linear readout of the reservoir state (Eq. (5)). On the other hand, as can be seen from the t-SNE analysis that **x**(*n*) states only partly contain the information of *u*(*n*−3) and *u*(*n*−4), the *r*^2^ value decreases with increasing *τ* when *τ* ≥ 3. This directly indicates the short-term memory property of the FeFET reservoir: the influence of the input history on the present state is strong for recent ones and becomes weaker for older ones. The short-term memory capacity, defined by ref. ^40^ ${C}_{{{{{\rm{STM}}}}}} = {{\sum}_{\tau = 1}^{\infty}}{r}^{2}(\tau)$, is estimated to be 2.35 without any explicit delay loops. As this reservoir computing system uses only a single FeFET, the memory capacity is attributed to the hysteretic and transient physical phenomena inside the FeFET.

We investigate the computing capability of nonlinear tasks by examining the temporal-XOR task^17^ and the parity-check task^20,41^. The temporal-XOR task, described by Eq. (12), requires to solve the XOR between the present input *u*(*n*) and the past input *u*(*n*−*τ*) (Fig. 4f for *τ* = 2), while the parity-check task, described by Eq. (13), requires to find out whether the total number of ones from *u*(*n*) to *u*(*n*−*τ*) is odd or even (Fig. 4j for *τ* = 2). Different from the delay task, these two tasks are linearly inseparable problems and require the reservoir part to have both short-term memory and nonlinearity. After training, the outputs *y*(*n*) corresponding to unseen inputs are shown in Fig. 4g for the temporal-XOR task (Fig. 4h after binarization) and in Fig. 4k for the parity-check task (Fig. 4l after binarization) with *τ* = 2. The high *r*^2^ values as well as the high accuracy >98% after binarization for the both tasks at *τ* ≤ 2 indicate that the FeFET-based reservoir computing system can be trained to compute these nonlinear tasks. The computing capability for *τ* ≥ 3 is limited by the short-term memory property of the reservoir since the tasks require the nonlinear interaction with the past inputs. The temporal-XOR capacity (*C*_XOR_) and the parity-check capacity (*C*_PC_), defined by the summations of *r*^2^ in the similar way to the short-term memory capacity^42^, are estimated to be 2.29 and 2.23, respectively. These *C*_XOR_ and *C*_PC_ values obtained from the single FeFET device are similar to those of the echo state network (neural network-based reservoir computing) with a size of 15 and 46 nodes, respectively (see Supplementary Fig. S9.1). This indicates that an FeFET is a potential solution for the hardware implementation of the reservoir part for solving nonlinear tasks without having to use recurrent neural networks with a vast number of connections. Note that the readout part has only linear operations (Eq. (5)) and thus the nonlinear property originates from the nonlinear dynamics in the FeFET. In comparison with the experimental demonstrations solving the parity-check task in systems using spin torque oscillators^43^ and a spin wave^16^, an FeFET-based reservoir computing system with a single-device reservoir can be implemented compactly in a CMOS-compatible manner without an external magnetic field or feedback loops.

**Fig. 5:**
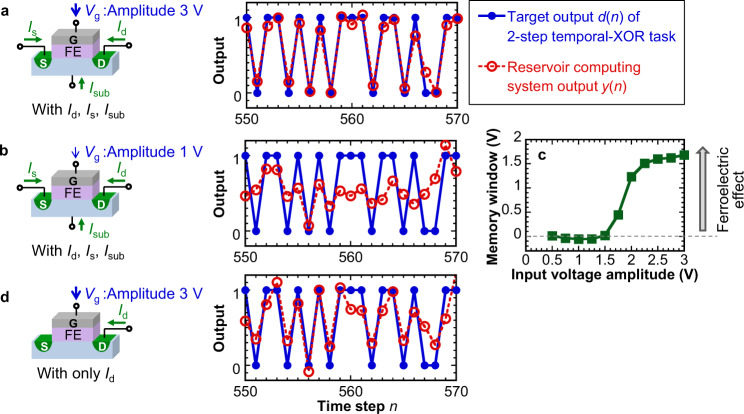
Comparison of reservoir computing system output for the 2-step temporal-XOR task under alternative operation schemes. **a**, **b** FeFETs under an input voltage amplitude of 3 V employed in this study (**a**) and under a lower amplitude of 1 V (**b**). **c** Memory window of FeFETs evaluated from the transfer characteristics. The ferroelectric behavior of the FeFET can be observed only when operated under an input voltage amplitude above 1.5 V, which is in agreement with the better performance of FeFET-based reservoir computing under higher input voltage amplitude. **d** Reservoir system in which only drain current of FeFET is employed in reservoir computing. The reservoir employing multiple current components consisting of drain current, source current, and substrate current of FeFETs (**a**) exhibits higher computing performance than that using only one single current component (**d**).

We examine the impact of the voltage amplitude of the mask function **v**(*n*), i.e., the maximum voltage *V*_g_ applied to the gate. As shown in Fig. 5a, b for the output time-series and in Supplementary Fig. S10.1 for the summary of the performance, the performance of FeFET-based reservoir computing systems substantially degrades when the gate voltage amplitude becomes lower than 1.5 V, which is also the minimum voltage amplitude to observe the ferroelectric hysteresis in the FeFET (Fig. 5c). Below this voltage, the electric field in the ferroelectric film is lower than the coercive field required to modulate the polarization states and thus the device operates similarly to a transistor with no ferroelectric functionality. The result implies that the FeFET can operate as a reservoir only when the polarization dynamics are driven by the voltage input, confirming the contribution of ferroelectric polarization to the reservoir computing capability.

The performance of the reservoir computing system using multi-terminal FeFETs is compared with the results when only the drain current *I*_d_(*t*) is used in reservoir computing. Figure 5a, d shows that utilizing currents from multiple terminals results in better computing performance. These current components are determined by different physical mechanisms in FeFETs (see Supplementary Fig. S5.1) and are equivalent to different nonlinear transformations. Hence, constructing the reservoir-state vectors by combining the *I*_d_(*t*), *I*_s_(*t*), and *I*_sub_(*t*) waveforms improve the dimensionality of the nonlinear transformation performed by the reservoir and results in higher reservoir computing performance. Moreover, we can change the bias voltage on the remaining terminals to further adjust the dynamics inside FeFETs for more efficient computing of given tasks, as demonstrated in Supplementary Fig. S11.1. Therefore, the multi-terminal structure of FeFETs is a remarkable feature that contributes to the high design flexibility of FeFET-based reservoir computing systems.

The above investigations revealed that a FeFET possesses the short-term memory and nonlinear transform capability to solve temporal nonlinear tasks. Here, we examine a more practical task by demonstrating the prediction of time-series output from a second-order nonlinear dynamical system. Among several possible second-order systems, we employ the system^44^ that has been previously studied^7,18,42^ (sometimes referred to as a NARMA2 system), in which the relation between the input *u*(*n*) and the model output *d*(*n*) of the system is described by

1 $$d(n)=0.4d(n-1)+0.4d(n-1)d(n-2)+0.6{u}^{3}(n)+0.1.$$

**Fig. 6:**
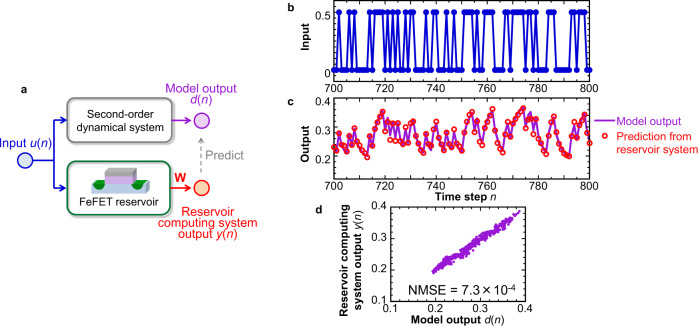
Performance of second-order nonlinear dynamic prediction task. **a** Schematic of second-order nonlinear dynamic task. The weight matrix **W** is trained so that the reservoir computing system output *y*(*n*) can well predict the model output *d*(*n*) of the second-order nonlinear dynamic system defined in Eq. (1). **b** Input signal *u*(*n*), which is a time-series of 0 and 0.5. **c** Prediction results *y*(*n*) of the second-order nonlinear dynamic system output by the trained FeFET reservoir computing system. A line shows the model output *d*(*n*) in Eq. (1). **d** Plot of the computed system output *y*(*n*) and the model output *d*(*n*). The relation that is mostly linear suggests the low prediction error by the trained reservoir computing system. NMSE, defined in Eq. (6), is found to be 7.3 × 10^−4^.

The weight matrix **W** is first trained by the ridge regression so that the output *y*(*n*) of the reservoir computing system follows *d*(*n*) of the second-order system in Eq. (1). Note that we employ 1-bit input in this demonstration (0 or 0.5; see Method). When an unseen data set is input after training, the output *y*(*n*) of the suitably-trained reservoir computing system is expected to be able to predict *d*(*n*) of the second-order system (Fig. 6a). For successful demonstration, the reservoir needs to have the internal state that reflects the nonlinear operation between the input, the 1-step-back output, and the 2-step-back output. This requires the reservoir part to have sufficient short-term memory capacity and nonlinearity.

Figure 6b, c show the input *u*(*n*) and output *y*(*n*) of the trained FeFET reservoir computing system. The theoretical model output *d*(*n*) is also shown as a solid line in Fig. 6c. The mostly linear plot between *d*(*n*) and *y*(*n*) in Fig. 6d confirms that the reservoir computing using the FeFET can predict the output of the second-order system with a small prediction error. The normalized mean squared error (NMSE) for the prediction is estimated to be 7.3 × 10^−4^, which is smaller by an order of magnitude than that obtained from a system without the FeFET (see Supplementary Fig. S12.1). Note that when using the training dataset as input (same dataset as that for training), the error *y*(*n*) − *d*(*n*) becomes smaller and NMSE becomes 6.4 × 10^−4^. These results on the second-order nonlinear dynamical task agree well with the results of the temporal-XOR and parity-check tasks in Fig. 4f–m: this reservoir computing system can perform the nonlinear operation using the information in the past.

## Discussion

We have demonstrated that a single-device FeFET can be utilized as a physical reservoir to realize a reservoir computing system in a compact manner. Due to the fact that the HfO_2_-based FeFET technology is highly compatible with the mature CMOS technologies, it is possible to extend the reservoir computing system by integrating multiple FeFETs in a circuit-level together with peripheral circuits. Such system is very useful because it allows us to further adjust the short-term memory capacity and the nonlinearity corresponding to computational tasks needed to be solved, as long as it satisfies the system area and power requirements. Supplementary Fig. S13.1 shows one demonstration of the reservoir system extension by connecting multiple FeFETs in parallel with delay operations, in which the prediction capability in the second-order nonlinear dynamical task can be significantly improved only by simple integration. The result clearly indicates a unique advantage: FeFET-based physical reservoir hardware has high design flexibility in adjusting its reservoir properties. Moreover, an array of multiple FeFETs allows parallel processing of reservoir computing for more efficient training and computational of complicated tasks^45^. Furthermore, a variety of designs for further extension of FeFET reservoir computing systems is feasible as HfO_2_-based FeFETs can be implemented by several approaches ranging from front-end-of-line^24^, back-end-of-line^46^ to 3D architectures^47^.

In summary, we have experimentally demonstrated reservoir computing using the FeFET with the Hf_0.5_Zr_0.5_O_2_ ferroelectric insulator as a CMOS-compatible physical reservoir for promising edge-AI computing hardware. We found that the FeFET has short-term memory and can nonlinearly transform the low-dimensional time-series inputs to the high-dimensional reservoir states, and thus can efficiently process time-series data to solve nonlinear tasks. The capability for reservoir computing of the FeFET is attributed to the unique dynamic properties of ferroelectric polarization and polarization/charge interaction, which have rarely been functionalized so far. The design flexibility originating from the FeFET’s various controllable parameters and the capability of circuit-level integration allows further adjustment of the reservoir properties according to target tasks, which contributes to not only flexible AI hardware but also to the progress of the fundamental understanding of physical reservoir computing. Due to the low training cost, FeFET-based reservoir computing is suitable for the processing of time-series data (for instance, speech recognition and data forecasting) at edge devices where the system needs to be adaptively updated in real-time according to the change in environment. FeFET-based reservoir computing systems, thanks to their CMOS compatibility and scalability, make it feasible to implement the reservoir computing function on a silicon chip with existing CMOS technologies as well as with FeFETs being employed in logic, memory, and computing-in-memory towards the next-generation intelligent systems.

## Methods

### Sample preparation

An FeFET with TiN/Hf_0.5_Zr_0.5_O_2_(10 nm)/SiO_2_(0.7 nm)/Si gate stack was fabricated by a gate-last process. A silicon substrate with a p-type doping concentration of 4 × 10^15^ cm^−3^ and highly n-doped source/drain regions was chemically oxidized by a standard hydrogen peroxide mixture at 70 °C for 90 s to form a SiO_2_ layer. Then, Hf_0.5_Zr_0.5_O_2_ was prepared by atomic layer deposition at 300 °C using tetrakis(ethylmethylamino)hafnium, tetrakis(ethylmethylamino)zirconium, and H_2_O as precursors, followed by a 16-nm TiN electrode by sputtering. Aluminum was deposited for gate and substrate contacts, and silicon-doped aluminum was deposited for source/drain contacts. The device was annealed at 400 °C for 30 s to form the ferroelectric phase in the Hf_0.5_Zr_0.5_O_2_ layer. The gate length and the gate width of the tested FeFET are 5 μm and 100 μm, respectively.

### Electrical measurements

The electrical characteristics were measured using a Keysight B1500A semiconductor device analyzer with B1530A waveform generator/fast measurement units. The B1530A units were connected to the gate of the FeFET for applying the gate voltage *V*_g_, to the drain for measuring the drain current *I*_d_(*t*) while applying a constant voltage of 300 mV, to the source for measuring the source current *I*_s_(*t*) while applying 0 V, and to the substrate for measuring the substrate current *I*_sub_(*t*) while applying 0 V. The applied voltage on the substrate was varied in Supplementary Section 11.

The time-series of binary inputs *u*(*n*) (*n* = 1, 2, …) were transformed to triangular voltage waveforms in the continuous-time domain *t* with an amplitude of 3 V, an offset of 0.5 V, and a time step of *T*_step_ = 4 μs. That is, masked signals corresponding to *u*(*n*) = 1 (or 0.5 for second-order nonlinear dynamic task) were swept from 0.5 V at *t* = *nT*_step_ to 3.5 V at *t* = (*n* + 1/2)*T*_step_ and back to 0.5 V at *t* = (*n* + 1)*T*_step_, and those corresponding to *u*(*n*) = 0 were swept from 0.5 V to −2.5 V and back to 0.5 V. The voltage amplitude was varied in the experiment investigating the impact of the amplitude. Each continuous-time waveform is expressed as an *M*-element (= 200-element) vector **v**(n), whose component *v*_*j*_(*n*) is the value at time *t* = (*n* + *j*/*M*)*T*_step_ (1 ≤ *j* ≤ *M*; *M* = 200). The masked signals were applied to the gate of the FeFET while the actual waveform of the applied voltage from the B1530A units were also monitored. All three currents were measured with *M* points per step, which corresponds to a sampling time of *T*_step_/*M* = 20 ns. A 3*M*-element (= 600-element) reservoir-state vector **x**(*n*) was constructed by the column-wise collection of *M*-element vectors **x**_**d**_(*n*), **x**_**s**_(*n*), and **x**_**sub**_(*n*), i.e., **x**(*n*) = [**x**_**d**_(*n*) | **x**_**s**_(*n*) | **x**_**sub**_(*n*)]. Here, the *j*th components of **x**_**d**_(*n*), **x**_**s**_(*n*), and **x**_**sub**_(*n*) are defined by the current values of *I*_d_(*t*), *I*_s_(*t*), and *I*_sub_(*t*) at time *t* = (*n* + *j*/*M*)*T*_step_ (1 ≤ *j* ≤ *M*), respectively.

### t-SNE analysis

We constructed the masked input waveforms **v**(*n*) and obtained the reservoir states **x**(*n*) corresponding to the binary inputs *u*(*n*) with a number of time step *n* of 4200. Each **v**(*n*) is treated as a 200-dimention data point and **x**(*n*) as a 600-dimention data point (thus, 4200 data points each for **v**(*n*) and **x**(*n*)). The t-SNE technique was used to reduce dimensions from 200 or 600 to 2 to visualize the scattering tendency of **v**(*n*) and **x**(*n*) in 2D plots. The perplexity of the t-SNE model was set to 30 and the early exaggeration factor was set to 15 for t-SNE analysis. The points in the t-SNE plot were labeled according to the input *u*(*n*) and the past input history *u*(*n*−1), *u*(*n*−2), *u*(*n*−3), and *u*(*n*−4).

### Evaluation of grouping tendency by *k*NN method

We separate the 4200 reservoir states **x**(*n*) randomly into 3780 for the training dataset and 420 for the testing data set. We labeled **x**(*n*) in the training dataset into 15 classes according to the input history as shown by the circled characters in Fig. 3c, and classified each **x**(*n*) in the testing dataset into one of the 15 classes by the *k*NN method with *k* = 9 (Choose 9 vectors in the training dataset that have shortest Euclid distances with the target vector, and classify to the most frequent class). The error rate is estimated by (number of **x**(*n*) that are incorrectly classified)/(total number of **x**(*n*)) with 10-fold cross-validation. Note that the error rate of this method when the 15 class labels are randomly shuffled (not following the grouping rule in Fig. 3c) is 93%.

### Training and testing of a reservoir system

10 sets of 1000 continuous steps of the binary input signals *u*(*n*) (=0 or 1, except for the second-order nonlinear dynamical task) were input through the mask function to the FeFET reservoir and the corresponding reservoir states **x**(*n*) were obtained experimentally. In each set, only the last 500 steps out of 1000 steps were used in the reservoir computing to avoid any impacts from the initial condition of the FeFET reservoir. 8 sets (4000 steps in total) were chosen as the training dataset and 2 sets (1000 steps in total) were chosen as the testing dataset, and cross-validation was carried out. By using the reservoir states **x**_**train**_(*n*) corresponding to the training dataset, the task-specific weight matrix **W** was trained by the ridge regression so that the outputs of the reservoir computing system

2 $${y}_{{{{{{\rm{train}}}}}}}(n)={{{{{{\bf{x}}}}}}}_{{{{{{\bf{train}}}}}}}(n)\cdot {{{{{\bf{W}}}}}}$$

becomes close to the target output *d*(*n*) of a given task (*y*_train_(*n*) and *d*(*n*): a number, which can be generalized to a row vector for multiple-output tasks; **x**_**train**_(*n*): 600-element row vector; **W**: 600 × 1 matrix). In particular, since ${y}_{{{{{{\rm{train}}}}}}}(n)={{{{{{\bf{x}}}}}}}_{{{{{{\bf{train}}}}}}}(n)\cdot {{{{{\bf{W}}}}}}$ has to satisfy for all *n*, we can summarize into one relation

3 $${{{{{{\bf{Y}}}}}}}_{{{{{{\bf{train}}}}}}}={{{{{{\bf{X}}}}}}}_{{{{{{\bf{train}}}}}}}{{{{{\bf{W}}}}}},$$

where **Y**_**train**_ = […, *y*_train_(*n*−1), *y*_train_(*n*), *y*_train_(*n* + 1), …]^⊤^ and **X** = […, **x**^⊤^_**train**_(*n*−1), **x**^⊤^_**train**_(*n*), **x**^⊤^_**train**_(*n* + 1), …]^⊤^ (**Y**_**train**_: *N* × 1 vector, where *N* is the total number of steps *n*; **X**_**train**_: *N* × 600 matrix). The optimum **W** is determined by using the training dataset through

4 $${{{{{\bf{W}}}}}}={({{{{{{\bf{X}}}}}}}_{{{{{{\bf{train}}}}}}}^{\top }{{{{{{\bf{X}}}}}}}_{{{{{{\bf{train}}}}}}}+\lambda {{{{{\bf{I}}}}}})}^{-1}{{{{{{\bf{X}}}}}}}_{{{{{{\bf{train}}}}}}}^{\top }{{{{{{\bf{Y}}}}}}}_{{{{{{\bf{train}}}}}}},$$

where **I** is the 600 × 600 identity matrix and *λ* is the ridge parameter. *λ* was optimized to be 8 × 10^−10^ A^2^ to minimize the test error. The trained weight matrix **W** was fixed during the testing procedure to calculate the system output

5 $$y(n)={{{{{\bf{x}}}}}}(n)\cdot {{{{{\bf{W}}}}}}$$

corresponding to the testing dataset. In this work, the generation of the voltage waveforms and the conversion to the reservoir states **x**(*n*) were carried out experimentally (see Methods-Electrical measurements), while the calculation of Eqs. (2)–(5) was performed in software as a demonstration.

The testing results were evaluated by several methods depending on the nature and the convention of corresponding tasks. In the second-order nonlinear dynamical task, NMSE was used for evaluating the error of the system output *y*(*n*) as compared to the target output *d*(*n*):

6 $${{{{{\rm{NMSE}}}}}}=\frac{{\sum }_{n}{({y}_{n}-{d}_{n})}^{2}}{{\sum }_{n}{d}_{n}^{2}}.$$

For the delay tasks, temporal-XOR tasks, and parity-check tasks, the squared correlation coefficient *r*^2^ between *y*(*n*) and *d*(*n*) (0 ≤ *r*^2^ ≤ 1) was used to evaluate the computing performance:

7 $${r}^{2}=\frac{{\sum }_{n}[({y}_{n}-\bar{y})({d}_{n}-\bar{d})]}{\sqrt{{\sum }_{n}{({y}_{n}-\bar{y})}^{2}{\sum }_{n}{({d}_{n}-\bar{d})}^{2}}}.$$

Here, $\bar{y}$ and $\bar{d}$ are the mean values of *y*_*n*_ and *d*_*n*_ over *n*, respectively. The short-term memory capacity, the temporal-XOR capacity, and the parity-check capacity are defined by the sums of *r*^2^ over different time delays:

8 $$C=\mathop{\sum }\limits_{\tau =1}^{\infty }{r}_{\tau }^{2}.$$

Here, ${r}_{\tau }^{2}$ denotes the squared correlation coefficient of tasks with a time delay *τ*.

In addition, based on the fact that the target outputs *d*(*n*) for the delay tasks, temporal-XOR tasks, and parity-check tasks are binary, the expected inference results can be estimated by binarizing the output through

9 $$B(y(n))=\left\{\begin{array}{ccc}0 & ; & y(n) \, < \,0.5\\ 1 & ; & y(n)\ge 0.5\end{array}.\right.$$

In this way, the error rate of the binarized results can be estimated by

10 $${{{{{\rm{Error}}}}}}\,{{{{{\rm{rate}}}}}}=\frac{{{{{{\rm{Number}}}}}}\,{{{{{\rm{of}}}}}}\,y(n)\,{{{{{\rm{that}}}}}}\,B(y(n))\,\ne \,d(n)}{{{{{{\rm{Total}}}}}}\,{{{{{\rm{number}}}}}}\,{{{{{\rm{of}}}}}}\,y(n)}.$$

### Delay, temporal-XOR, and parity-check tasks

The target outputs *d*(*n*) of the delay task (sometimes referred to as the short-term memory task), temporal-XOR task, and parity-check task for a given delay step *τ* can be expressed by

11 $$d(n)=u(n-\tau ),$$

12 $$d(n)=u(n)\oplus u(n-\tau ),$$

13 $$d(n)=u(n)\oplus u(n-1)\oplus ...\oplus u(n-\tau )=\mathop{\sum }\limits_{l=0}^{\tau }u(n-l)({{{{\mathrm{mod}}}}}\,2),$$

respectively. Here, ⊕ represents the XOR operator. We ran these tasks within the 1 ≤ *τ* ≤ 10 range and confirmed that the squared correlation coefficients *r*^2^ fall to almost zero at *τ* ≤ 10. We also carried out the task in Eq. (11) when *τ* = 0 to confirm that the trained reservoir computing system can generate the input *u*(*t*).

### Input of second-order nonlinear dynamical task

We examined the second-order nonlinear dynamical task by considering binary inputs similarly to other tasks in this Article. Since the inputs of this task must be within the [0, 0.5] range^44^, we considered scaled binary inputs: *u*(*n*) = 0 or 0.5. The same mask function was used. That is, the masked signals are +3 V triangular waveforms for *u*(*n*) = 0.5 and −3 V triangular waveforms for *u*(*n*) = 0 with an offset of 0.5 V. The dataset construction and training procedure were the same as other tasks.

### Supplementary information

Toprasertpong_PR File

Supplementary Information

## Data Availability

We have uploaded the source data to the Zenodo database, accessible at: 10.5281/zenodo.6816576. The data that support the findings of this study are available from the corresponding author upon reasonable request.
