# Supplementary material for: Reservoir computing on a silicon platform with a ferroelectric field-effect transistor
Source: Commun Eng. 2022 Aug 5;1:21. doi: 10.1038/s44172-022-00021-8 (PMC10956125; doi:10.1038/s44172-022-00021-8)
Supplement: Supplementary file 2 — Supplementary Information [file 44172_2022_21_MOESM2_ESM.pdf]

Supplementary Material

Reservoir computing on a silicon platform with a ferroelectric field-effect transistor

Kasidit Toprasertpong[1,2]*, Eishin Nako[1,2], Zeyu Wang[1], Ryosho Nakane[1], Mitsuru Takenaka[1] and Shinichi Takagi[1]

[1]*Department of Electrical Engineering and Information Systems, The University of Tokyo, 7-3-1 Hongo, Bunkyo-ku, Tokyo 113-8656, Japan*
[2]*These authors contributed equally to this work.*

***Corresponding author:** toprasertpong@mosfet.t.u-tokyo.ac.jp

Supplementary Section 1: Schematic of reservoir computing

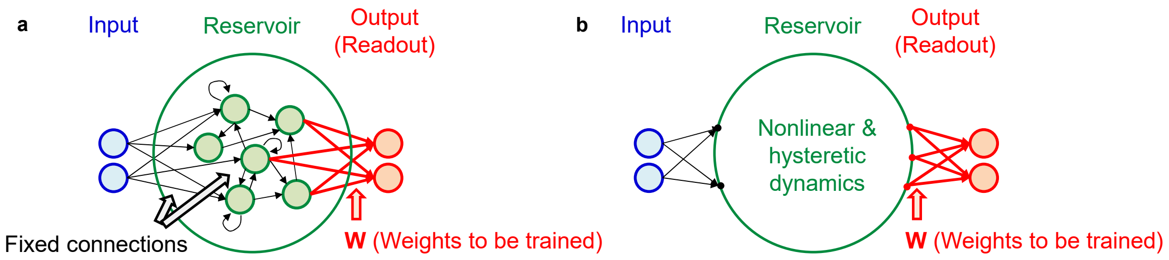

Supplementary Fig. S1.1 | Schematic of reservoir computing. a, Eco state network, or reservoir computing whose reservoir part is a recurrent neural network. Most reservoir computing systems performed in software employ this type of reservoir computing. **b,** Physical reservoir computing. The reservoir part is implemented by a physical system having nonlinear and hysteretic dynamics. The computing performance relies on the short-term memory (capability to hold the information of recent input history in the reservoir) and the nonlinear dynamics (capability to nonlinearly transform inputs with a low dimension to the reservoir states with a higher dimension) of the reservoir. In both types of reservoir computing, the reservoir states are readout by one-layer connection with a weight matrix **W**.

Supplementary Section 2: FeFET structure and device characteristics

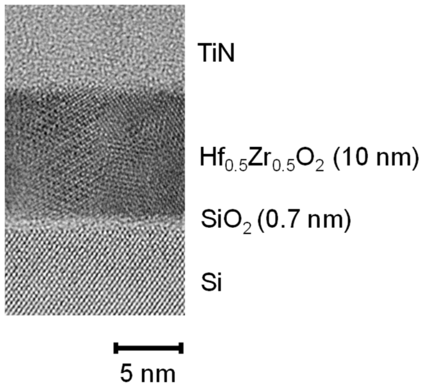

Supplementary Fig. S2.1 | Transmission electron microscope (TEM) of gate stack structure. The gate stack structure of the FeFET consists of TiN/$Hf_{0.5}Zr_{0.5}O_2$ (10 nm)/SiO_2 (0.7 nm)/Si. The $Hf_{0.5}Zr_{0.5}O_2$ layer shows a polycrystalline structure, indicating the successful crystallization to the ferroelectric phase (orthorhombic phase) after the annealing process at 400°C for 30 s.

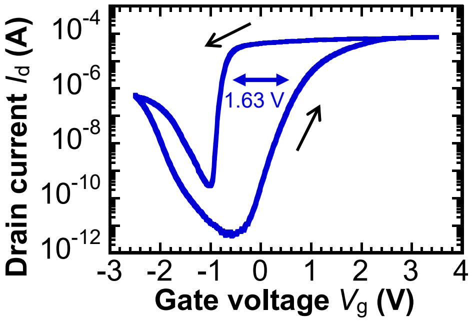

Supplementary Fig. S2.2 | I_d-V_g characteristics and memory window. The I_d-V_g characteristics were measured in a quasi-static manner. The ferroelectric-type hysteresis has been confirmed. That is, the threshold voltage V_{th} is shifted to the negative direction after the gate is applied with positive voltage V_g of 3.5 V, and V_{th} is shifted to the positive direction after being applied by negative gate voltage V_g of -2.5 V. The memory window, defined by the gate voltage difference at the current level of (100 nA)×W/L = 2 µA (W = 100 µm, L = 5 µm), is 1.63 V.

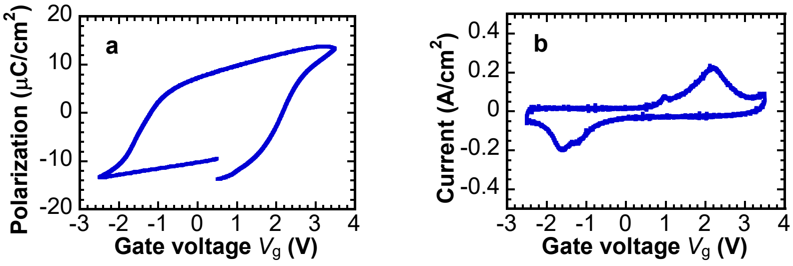

Supplementary Fig. S2.3 | Polarization properties of the FeFET. a, Polarization-gate voltage (P-V_g) characteristics of the FeFET measured by the method in Ref. [1] at 1 kHz. **b,** Current obtained during the polarization measurement. The ferroelectric hysteresis and the polarization switching current peaks have been clearly observed, confirming the ferroelectric properties of the FeFET in this work.

Supplementary Section 3: History-dependent ferroelectric state

Supplementary Fig. S3.1a depicts an example showing that the polarization state P of a ferroelectric material even under the same input is dependent on the input history. With the same electric field $E(n)$ at the present step n but different electric field $E(n\text{-}1)$ in the previous step, the polarization state follows different trajectories in the hysteresis curve and thus exhibits different polarization states. The impact of input history can also be roughly explained by the static Preisach model[2] as shown in Supplementary Figs. S3.1b-c. These illustrations show that the polarization state at the present step $P(n)$ is dependent on not only $E(n)$ and $E(n\text{-}1)$ but also on the history further back in the past such as $E(n\text{-}2)$ and so on. Note that the Preisach model is just a simplified example (assuming static condition) to help roughly understand the short-term memory behavior in ferroelectric materials. In actual operations, transient dynamics make the situation more complicated than the Preisach model even for binary inputs.

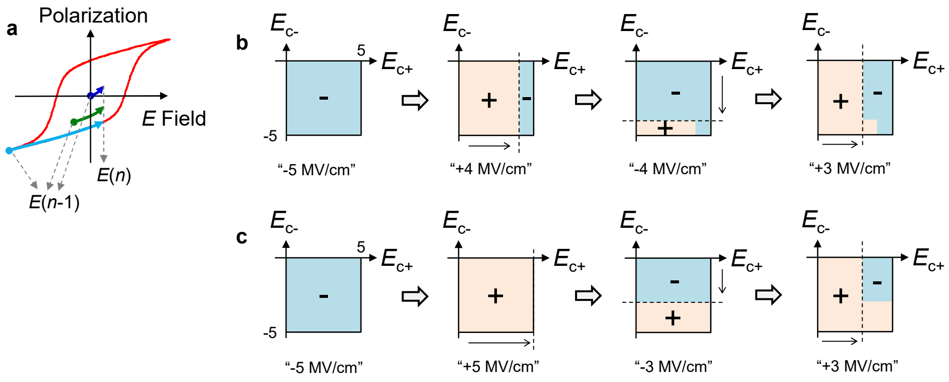

Supplementary Fig. S3.1 | History-dependent polarization state in ferroelectric materials. a, Hysteresis property of ferroelectric materials showing that the polarization state $P(n)$ at step n under the same applied field $E(n)$ may be different depending on the field $E(n\text{-}1)$ at the previous step. **b,c,** Polarization states described by the Preisach model. Each point on the graph corresponds to the polarization domain with the positive coercive field E_{c+} and the negative coercive field E_{c-}. The orange/blue areas correspond to the positive/negative polarization states of domains with given (E_{c+}, E_{c-}). The electric field is applied in the order of -5 MV/cm → +4 MV/cm → -4 MV/cm → +3 MV/cm for (**b**) and -5 MV/cm → +5 MV/cm → -3 MV/cm → +3 MV/cm for (**c**). These examples suggest that the input history further than 2 steps back in the past has an influence on the present polarization state.

Supplementary Section 4: Nonlinear dynamics in an FeFET

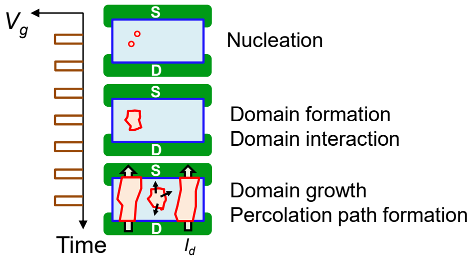

Supplementary Fig. S4.1 | Top-view schematic of polarization dynamics in an FeFET. Under applied gate voltage V_g, electric field drives a variety of dynamics inside the ferroelectric insulator. This includes domain nucleation and formation, which is known to have an accumulative behavior (domain formation occurs only after accumulated input is over a certain voltage/time limit[3]), interaction between domains, and domain growth. The channel current flows only through the percolation paths which are distributed according to the polarization distribution and the polarization/charge interaction. The different time scales of each dynamic results in nonlinear response to the input.

Supplementary Section 5: Current components in an FeFET

Supplementary Fig. S5.1 shows the main current components flowing in an FeFET. The channel current I_{ch}, flowing from the drain to the source, originates from the flow of mobile charges in semiconductor driven by the drain voltage and is the main component during the ON-state operation of FeFETs. I_{ch} is approximately proportional to the mobile charge density, which is determined by the difference between the ferroelectric polarization P and trapped charges Q_{trap} in the gate stack[4]. The capacitive current I_{cap}, or the charging/discharging current, originates from the movement of charges dQ/dt to charge/discharge the gate capacitor when the applied voltage changes with time, and is strongly induced by the transient dynamics of polarization dP/dt. For the inversion-mode FeFET in this work, the majority-carrier component $I_{cap\text{-}major}$ flows through the substrate, and the minority-carrier component $I_{cap\text{-}minor}$ flows through the source/drain. The capacitive current is affected by the operating frequency as well as the device area, so these parameters also have to be carefully designed in order to efficiently control each current component of an FeFET.

As the drain, source, substrate currents can be expressed by

$$I_d = I_{\text{ch}} - I_{\text{cap-minor}}, \quad (1)$$

$$I_s = -I_{\text{ch}} - I_{\text{cap-minor}}, \quad (2)$$

$$I_{\text{sub}} = -I_{\text{cap-major}}, \quad (3)$$

measuring all three currents provides different information of ferroelectric dynamics and helps us readout various dynamics of ferroelectric polarization. Note that there are also other extrinsic current components that are not mentioned here but can appear in actual devices such as leakage current, which are also controlled by the dynamics of polarization P.

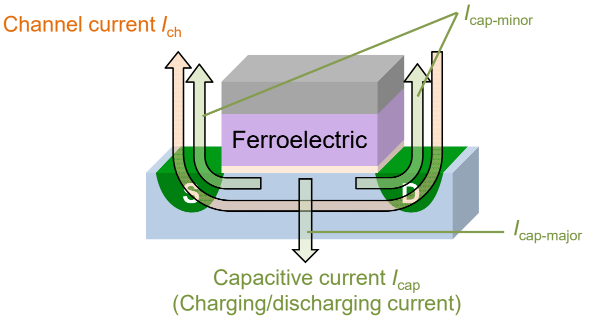

Supplementary Fig. S5.1 | Main components of current detected from the drain, source, and substrate of an FeFET. The channel current I_{ch}, driven by the drain voltage, and the capacitive current I_{cap}, driven by the time-derivative of gate voltage, are the main current components in an FeFET. In an inversion-mode FeFET, the capacitive current flows through the substrate ($I_{cap\text{-}major}$) when the flowing charges are the majority carrier and through the source/drain when the flowing charges are the minority carriers ($I_{cap\text{-}minor}$).

Supplementary Section 6: Nonideality of measurement instrument

Considering that the applied voltage V_g to the gate of FeFET are given by the masked input waveform $\mathbf{v}(n)$, and that the masked input waveform $\mathbf{v}(n)$ has the same signal dimension as the original input $u(n)$ (see Fig. 3a,b in the main text), we expect that the gate voltage V_g should have the same dimension as the input $u(n)$. In other words, the input preprocessing itself should not contribute to the high-dimensional transformation and thus the reservoir computing performance. However, in actual experiment, it may deviate from the ideal situation as the actual voltage V_g was generated by the power source of nonideal measurement instrument. Supplementary Figs. S6.1a-b compare the programmed V_g defined by the mask input waveform $\mathbf{v}(n)$ and the actual V_g monitored during the measurement. We can see that the measurement instrument can well reproduce the shape of the masked input waveform $\mathbf{v}(n)$, but with very small distortion due to the instrument finite bandwidth.

To examine the impact of this nonideality of input signals, we consider the actual voltage waveforms $\mathbf{V_g}(n)$ (200-element vectors whose jth element is the actual V_g monitored at time $(n+j/M)T_{\mathrm{step}}$) and analyze with t-SNE as shown in Supplementary Fig. S6.1c. Different from the programmed V_g waveforms (= masked input waveforms $\mathbf{v}(n)$) exhibiting only two points in the t-SNE map as shown in Fig. 3b in the main text, the actual waveforms $\mathbf{V_g}(n)$ become four clusters in the t-SNE map. This implies that even though the distortion of voltage waveforms is very small when looking at Supplementary Figs. S6.1a-b, this nonideality of the measurement instrument unintentionally increases the dimension of the input signal and thus the careful interpretation of the results is needed. We can see from the t-SNE map that the clusters are formed according to the values of $u(n)$ and $u(n\text{-}1)$. That is, the actual input waveforms V_g are distorted depending on the value of the previous input and thus they contain the information of $u(n\text{-}1)$.

To investigate the impact on the reservoir computing performance, we replace $\mathbf{x}(n)$ in reservoir computing with $\mathbf{V_g}(n)$ so that the system output is given by $y(n) = \mathbf{V_g}(n)\cdot\mathbf{W}$. This is approximately equivalent to removing the FeFET from the reservoir computing system. Supplementary Figs. S6.1d-f show the computing performance, evaluated by the squared correlation coefficient with the target output, of delay tasks, temporal-XOR tasks, and parity-check tasks after replacing $\mathbf{x}(n)$ with $\mathbf{V_n}(n)$. This computing system can be performed even without the FeFET for the 1-step delay task (task estimating $u(n\text{-}1)$) as can be expected from the fact that the distortion of the actual waveform $\mathbf{V_n}(n)$ is dependent on the previous input $u(n\text{-}1)$. On the other hand, it shows that $\mathbf{V_n}(n)$ cannot compute tasks with longer delay as well as nonlinear tasks, suggesting that the input distortion has only small effect on these tasks. In this way, we can say that the performance of the FeFET-based reservoir computing system in nonlinear tasks is attributed to the dynamics in the FeFET. For delay tasks, the contribution of nonideality of the measurement instrument partly involves, but we can still say that the result of the short-term memory capacity in this work cannot be achieved without the FeFET. It should be noted that the distortion of the input waveforms becomes more apparent for shorter input time step T_{step} because of the instrument finite bandwidth. We chose $T_{\mathrm{step}} = 4$ μs in this work to minimize the effect of the waveform distortion.

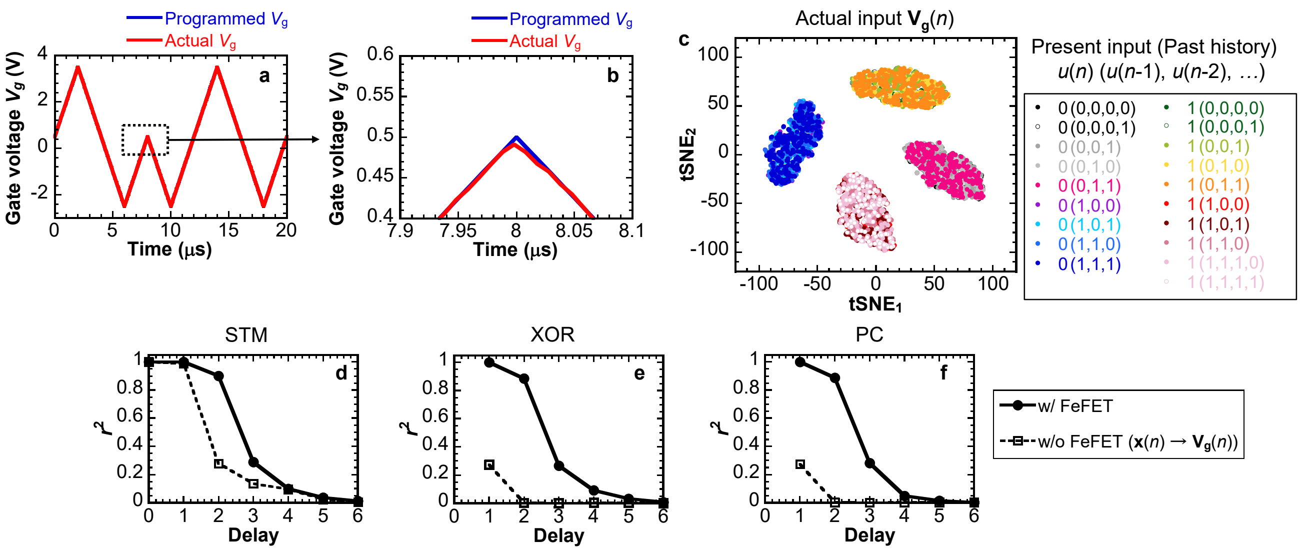

Supplementary Fig. S6.1 | Impact of nonideality of measurement instrument on the reservoir states and reservoir computing performance. a, Programmed and actual V_g waveforms. The programmed V_g is defined by the masked input waveform $\mathbf{v}(n)$. The actual V_g was monitored during the measurement by Keysight B1530A waveform generator/fast measurement units. **b,** Zoomed-in waveforms. Slight distortion was observed when the voltage slope changes rapidly. **c,** t-SNE map of 200-element vectors $\mathbf{V}_g(n)$. **d-f,** Squared correlation coefficient r^2 between the computed output signal and the target signal of delay tasks (**d**), temporal-XOR tasks (**e**), and parity-check tasks (**f**) when the reservoir states $\mathbf{x}(n)$ are replaced by the actual voltage waveforms $\mathbf{V}_g(n)$ applied to the FeFET gate to examine the impact of measurement instrument. The solid lines are the results with the FeFET (using $\mathbf{x}(n)$) shown in Fig. 4 in the main text.

Supplementary Section 7: Comparing reservoir states x(n) for different u(n-2)

Figure 2 in the main text shows 8 reservoir states depending on $u(n)$, $u(n\text{-}1)$, and $u(n\text{-}2)$. Even though the states with the same $u(n\text{-}2)$ look similar, they can be distinguishable by t-SNE (Fig. 3c) and by the reservoir computing in the 2-step delay task (Fig. 4c). As shown in Supplementary Fig. S7.1, the difference between $\mathbf{x}(n)$ with different $u(n\text{-}2)$ is small but significant enough that can be distinguished during computing.

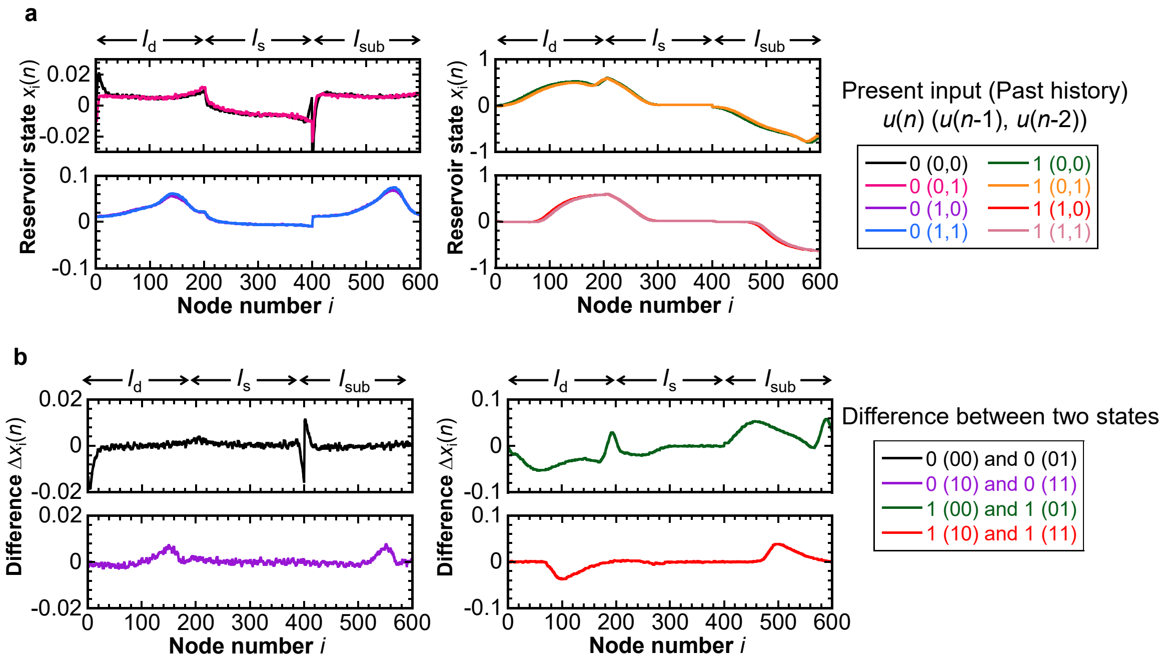

Supplementary Fig. S7.1 | Close comparison of internal states of FeFET reservoir. a, Reservoir states $\mathbf{x}(n)$ corresponding to the same $u(n)$ and $u(n\text{-}1)$ but different $u(n\text{-}2)$ are shown in the same plot. **b,** Difference $\Delta\mathbf{x}(n)$ of the states with the same $u(n)$ and $u(n\text{-}1)$. Even though the shape may look similar, small difference can be observed, which can be clearly distinguished by t-SNE (Fig. 3c) as well as by the readout of reservoir computing (Fig. 4c).

Supplementary Section 8: Ridge regression

The ridge regression

$$\mathbf{W} = (\mathbf{X}_{\mathbf{train}}^{\top}\mathbf{X}_{\mathbf{train}} + \lambda\mathbf{I})^{-1}\mathbf{X}_{\mathbf{train}}^{\top}\mathbf{Y}_{\mathbf{train}} \quad (4)$$

is used to determine the weight matrix **W** in the training process. The ridge parameter λ helps prevent overfitting of the model. Supplementary Fig. S8.1 shows the short-term memory capacity C_{STM}, temporal-XOR capacity C_{XOR}, and the parity-check capacity C_{PC} for different λ. It can be seen that, mostly independent of tasks, λ that gives the maximum performance is around 8×10^{-10} A^2. We use this value throughout this work.

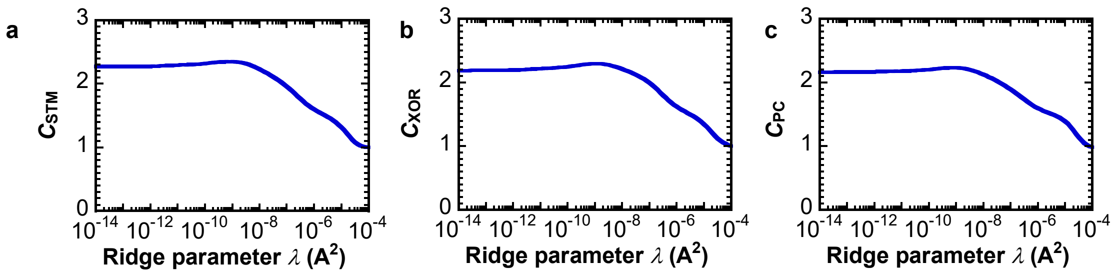

Supplementary Fig. S8.1 | Impact of ridge parameter on the reservoir performance. a, Short-term memory capacity C_{STM}. **b,** Temporal-XOR capacity C_{XOR}. **c,** Parity-check capacity C_{PC}. All tasks considered here reach the maximum performance at λ around 8×10^{-10} A^2.

We further investigate the role of λ by examining the trained **W** as shown in Supplementary Fig. S8.2. When the ridge regression is not used (when $\lambda = 0$; also called linear regression), the weight elements look noisy, which is attributed to the overfitting to specific inputs. On the other hand, when the ridge regression is used, the trained **W** tends to follow the overall trend rather than following individual inputs. However, when λ is too large, underfitting occurs and the trained weights loss the essential information for reservoir computing. It is worth noting that the trend of weight matrix elements W_{i} looks similar to the smooth processing of data with noise: the noise is smoothened when λ increases, and becomes over-smoothened when λ becomes too large. From Supplementary Fig. S8.2, the optimal value of λ is found to be between 10^{-10} to 10^{-8} A^2, which is the range of $\lambda = 8\times10^{-10}$ A^2 that we use in this work.

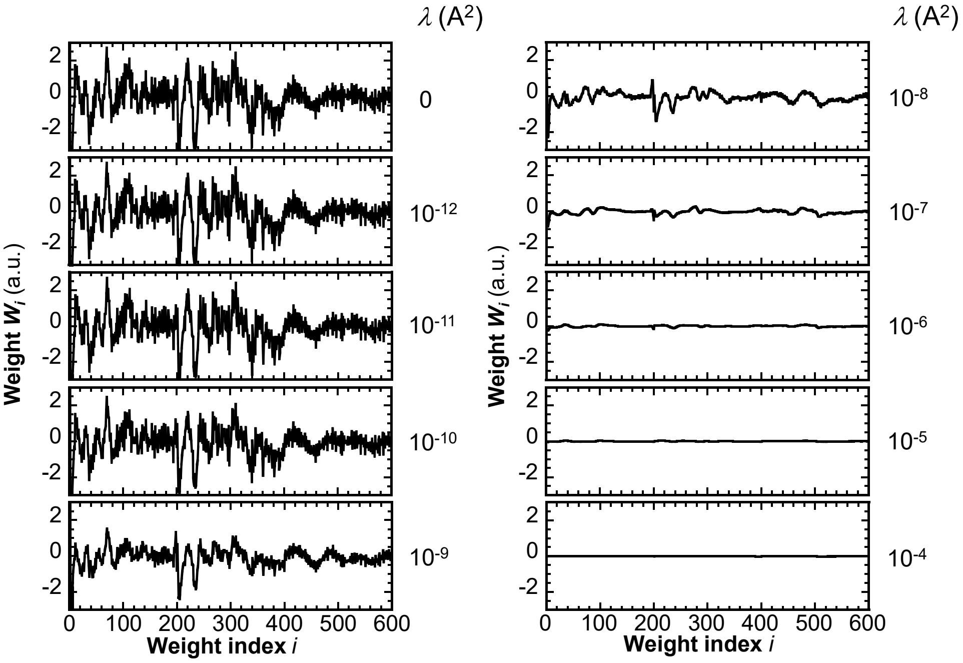

Supplementary Fig. S8.2 | Trained weights after ridge regression. The weight matrix element W_i of the 2-step delay task is plotted for different ridge parameters λ. Noisy W_i and over-smoothened W_i are confirmed for too small λ and too large λ, corresponding to the model overfitting and underfitting behaviours, respectively.

Supplementary Section 9: Comparison with echo state networks

We make a comparison with echo state networks, which are reservoir computing typically employed in software, to get a clearer picture of the performance of the FeFET-based reservoir computing. The schematic of echo state networks is shown in Supplementary Fig. S9.1a. Echo state networks are a classical type of reservoir computing whose reservoir part is implemented by recurrent neural networks with predetermined weights, represented by a matrix $\mathbf{W}_{\mathbf{res}}$. $\mathbf{W}_{\mathbf{res}}$ used in this Section is a sparse matrix whose elements are randomly fixed to zero with a probability of 0.9 (10% connection). Nonzero elements are determined by a uniform distribution on [-1,1] and are subsequently scaled so that the spectral radius of the matrix becomes 0.9. The activation function is the tanh function similarly to that used in most eco-state networks. $\mathbf{W}_{\mathbf{in}}$ is a matrix connecting the input and the reservoir and its elements are determined by a uniform distribution on [-1,1]. $\mathbf{W}_{\mathbf{res}}$ and $\mathbf{W}_{\mathbf{in}}$ are randomly determined by the mentioned distributions and are fixed (not updated by training) in each run of simulation. In each simulation run, 5000 steps of binary inputs are used for training and 3000 steps are used for testing. Before each training and testing, the reservoir is run in advance by 1000 steps to avoid the effect of the initial condition of the reservoir states $\mathbf{x}(n)$. The performance was averaged over 200 simulation runs.

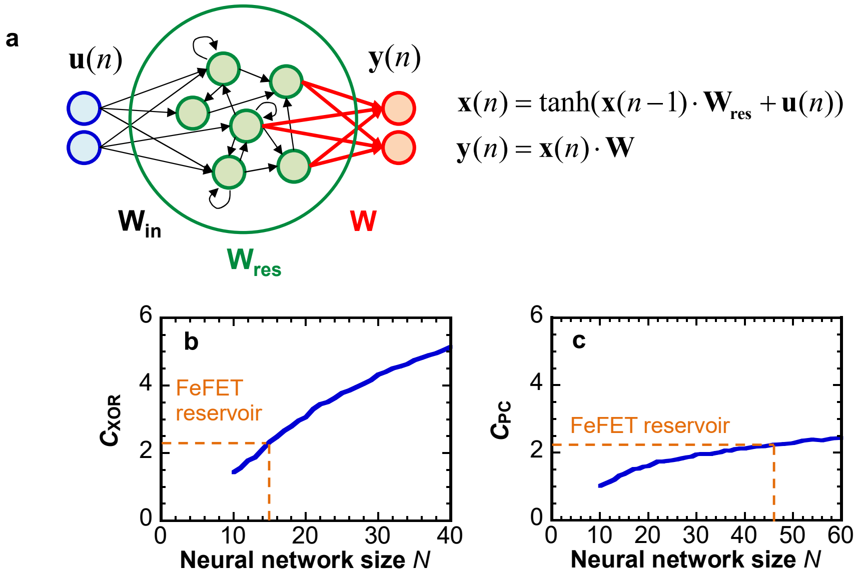

Supplementary Fig. S9.1 | Performance of temporal-XOR task and parity-check task in eco state networks. a, Schematic of an eco state network. $\mathbf{W}_{\mathbf{in}}$ and $\mathbf{W}_{\mathbf{res}}$ are fixed and $\mathbf{W}$ is trained corresponding to each specific task. The tanh activation function is used. **b-c,** Temporal-XOR capacity (**b**) and parity-check capacity (**c**) for eco state networks with different reservoir sizes N. The FeFET-based reservoir computing system shows C_{XOR} = 2.29 and C_{PC} = 2.23, which has similar performance to the eco-state network with N = 15 and N = 46, respectively.

We compare the computing performance of the FeFET-based reservoir computing with echo state networks in the temporal-XOR tasks and the parity-check tasks. The temporal-XOR capacity C_{XOR} and the parity-check capacity C_{PC} of the echo state networks with different network size N (size of

$\mathbf{W_{res}}$) are shown in Supplementary Figs. S9.1b-c. The FeFET-base reservoir computing system in this work, having C_{XOR} = 2.29 and C_{PC} = 2.23, has the similar performance to the echo state network with the size of 15 and 46, respectively. These results indicate that the FeFET exhibits satisfactory nonlinear dynamics and is promising for the implementation as a physical reservoir. If an echo state network with a size of N = 46 has to be implemented by hardware, a system with 46 nodes with 212 random connections (10% of 46×46 possible connections) is required. On the other hand, a reservoir computing system with similar computing performance is achieved only by a single FeFET device.

The equivalent sizes of the echo state network are not the same for different tasks because of the different dynamics in the FeFET and echo state networks, implying that the design strategy in echo state networks may only partly applicable to physical reservoirs, and thus further understanding of required properties of physical reservoirs is needed. High design flexibility of FeFET is expected to contribute to a fundamental study in the field of physical reservoirs.

Supplementary Section 10: Impact of input voltage amplitude

The actual input signal is converted by the mask function to the voltage waveform to be applied to the gate of FeFET as gate voltage V_g during the reservoir computing operation. The masked waveform $\mathbf{v}(n)$ in this study is a triangular waveform with a voltage amplitude of 3 V and an offset of 0.5 V. To investigate the impact of input voltage, the reservoir computing performance under different input voltage amplitudes (different mask functions) varied from 0.25 V to 3 V is examined as shown in Supplementary Fig. S10.1. The short-term memory capacity C_{STM}, the temporal-XOR capacity C_{XOR}, and the parity-check capacity C_{PC} significantly decrease when the input voltage amplitude is below 1.5 V. As shown in Fig. 5c in the main text that the ferroelectric memory window in FeFETs can be observed when the input voltage amplitude is above 1.5 V, the similar tendency between the ferroelectric behavior and the reservoir computing performance implies that the reservoir computing capability is attributed to the ferroelectric polarization in FeFETs.

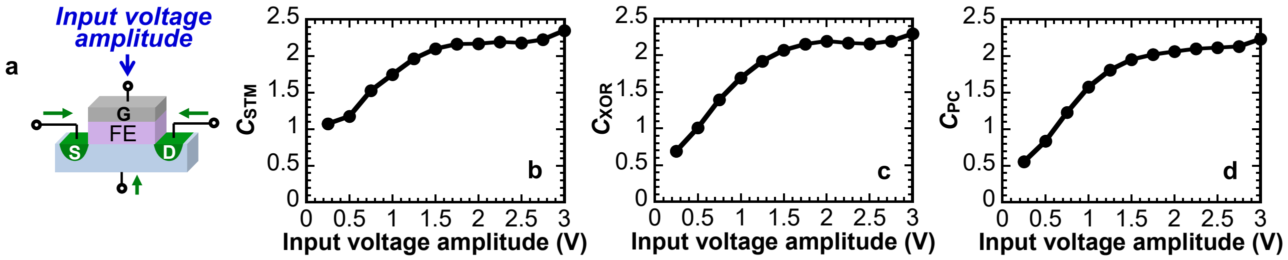

Supplementary Fig. S10.1 | Performance of reservoir computing using an FeFET operated under different input voltage amplitudes. a, Operation schematic. **b,** Short-term memory capacity C_{STM}. **c,** Temporal-XOR capacity C_{XOR}. **d,** Parity-check capacity C_{PC}. All the performances decrease with decreasing input voltage amplitude because of the smaller contribution of the ferroelectric polarization dynamics.

Supplementary Section 11: Reservoir tunability by substrate bias

As a demonstration of the design flexibility of reservoir computing using an FeFET with multiple terminals, we examine the reservoir computing performance under different substrate biases. Supplementary Fig. S11.1 shows C_{XOR} from the temporal-XOR task, C_{PC} from the parity-check task, and NMSE results from the second-order nonlinear dynamical task under the substrate biases varied from 0 V to -5 V. We can see that substrate biases that give the maximum performance is task-dependent: -2.25 V for the temporal-XOR, 0 V for the parity-check task, and -5 V for the second-order nonlinear dynamical tasks. The difference in optimum points is due to the fact that different tasks have different computational complexity and may require different properties of the reservoir to solve. By applying the FeFET substrate bias, the depletion region in the semiconductor channel is modulated, affecting the dynamics of the drain current, source current, and substrate current. This indicates that the multi-terminal feature of FeFETs enables fine-tuning of the FeFET reservoir to handle different tasks effectively with the same devices and hardware configurations. This feature would be beneficial in practical implementation in which the hardware cannot be reconstructed after it is physically integrated into a chip.

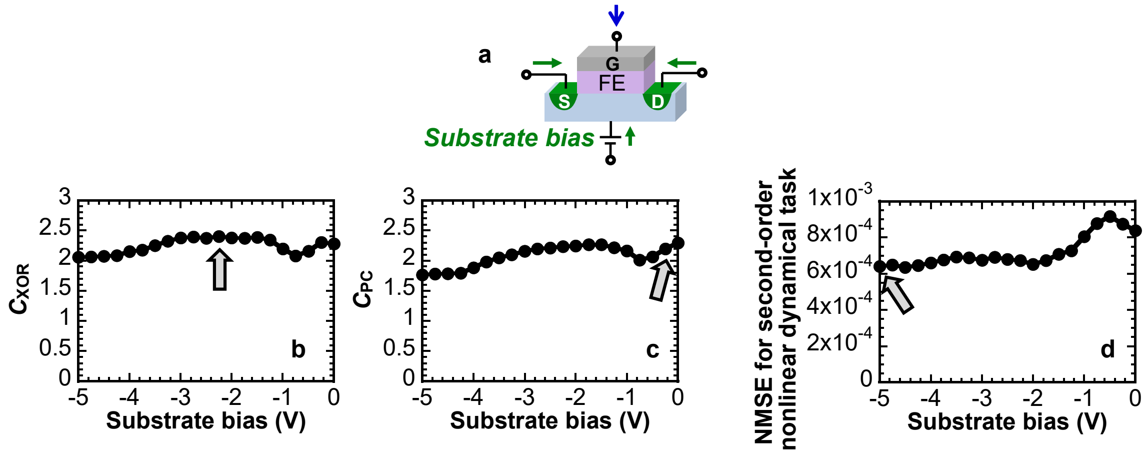

Supplementary Fig. S11.1 | Tunable reservoir properties of FeFETs by substrate bias. a, Operation schematic. **b,** Temporal-XOR capacity C_{XOR}. **c,** Parity-check capacity C_{PC}. **c,** NMSE for second-order nonlinear dynamical task. Note that high C_{XOR}, high C_{PC}, and low NMSE correspond to higher performance. Within the -5 V ≤ substrate bias ≤ 0 V range with a 0.25 V measurement step, the optimal substrate biases are -2.25 V, 0 V, and -5 V for the temporal-XOR, parity-check, and second-order nonlinear dynamical tasks, respectively, as indicated by the arrows in the figures.

Supplementary Section 12: Contribution of the FeFET in the second-order nonlinear dynamical task

To investigate the contribution of the FeFET in the second-order nonlinear dynamical task, we try evaluating the reservoir computing system when the FeFET is removed. Let's first consider a system where the masked input waveform $\mathbf{v}(n)$ is directly readout $y(n) = \mathbf{v}(n)\cdot\mathbf{W}$ instead of the reservoir state $\mathbf{x}(n)$ in both the training and testing procedures as shown in Supplementary Fig. S12.1a. We can see that the output $y(n)$ of this system without the FeFET cannot well predict the model output $d(n)$ expected from the second-order nonlinear dynamical system. Despite this fact, the value of NMSE = 8.9×10^{-3} in this case is apparently low due to the limited range of $y(n)$, suggesting that the interpretation of the absolute value of NMSE has to be carefully performed. We also consider the influence of the measurement instrument (see Supplementary Section 6) by reading out from the distorted gate voltage waveform $\mathbf{V}_\mathbf{g}(n)$ and carry out $y(n) = \mathbf{V}_\mathbf{g}(n)\cdot\mathbf{W}$. Supplementary Fig. S12.1b shows that the capability to predict the second-order nonlinear dynamical system is improved and the NMSE reduces to 4.3×10^{-3} owing to the small short-term memory of $\mathbf{V}_\mathbf{g}(n)$. On the other hand, the reservoir computing system with the FeFET in Supplementary Fig. S12.1c shows the satisfactory prediction capability (improved linear relation between $y(n)$ and $d(n)$) and an order of magnitude improvement of NMSE, indicating that the performance of the second-order nonlinear dynamical task is the contribution of the FeFET.

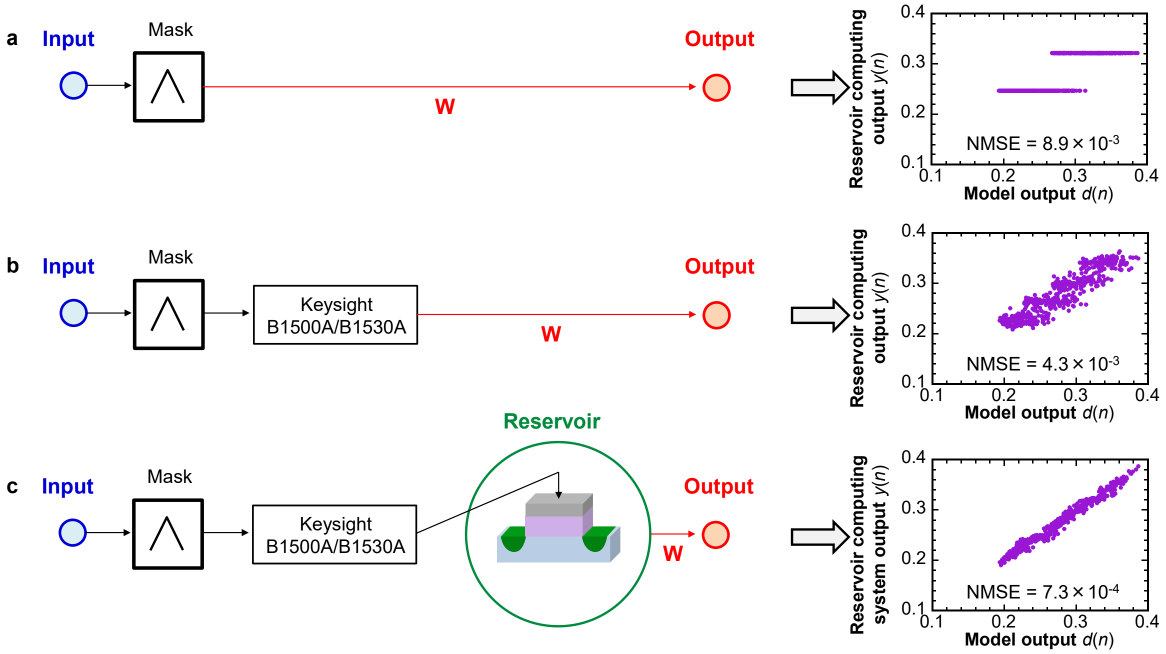

Supplementary Fig. S12.1 | Second-order nonlinear dynamical task with and without the FeFET. a, Direct training/testing from the masked input. **b,** System where the FeFET is removed but the effect of measurement instrument is still taken into account. **c,** Reservoir computing using the FeFET. Plots on the right show the relation between the ideal output of the second-order nonlinear dynamical system $d(n)$ and the output predicted by the reservoir computing systems $y(n)$. NMSEs evaluated by predicting the unknown dataset after training are 8.9×10^{-3}, 4.3×10^{-3}, and 7.3×10^{-4}, indicating that the dynamics in FeFET is the main contribution for performing this task.

Supplementary Section 13: Reservoir computing system using multiple FeFETs

As FeFETs are CMOS-compatible electron devices, we can take this advantage by integrating multiple FeFETs electrically to enhance their functionality. Supplementary Fig. S13.1a shows one simple example that uses multiple FeFETs and delay operations. The input time-series with delays of i steps ($0 \leq i \leq D$-1), $u(n$-$i)$, are preprocessed (masked and converted to voltage waveforms) and input to separate FeFET reservoirs. These D sets of FeFET reservoirs, the original state of which has M elements each, are connected in parallel to form the reservoir state represented by a $D{\cdot}M$-element vector. The combined reservoir state is eventually readout through a $D{\cdot}M{\times}1$ weight matrix $\mathbf{W}$. To make an estimation of the performance of this parallel system, we obtained the reservoir-state vector $\mathbf{x}(n)$ of a single FeFET experimentally, formed a new reservoir-state vector [$\mathbf{x}(n)$, $\mathbf{x}(n$-1), …, $\mathbf{x}(n$-(D-1))], and operated this reservoir computing by simulation.

The memory capacity C_{STM} of this system is shown in Supplementary Fig. S13.1b. Since the information of the past history is intentionally included in the reservoir states, C_{STM} is increased after inserting more delay operations as expected. This indicates that, even though C_{STM} of an FeFET is around 2.3, it can be easily improved in a circuit level using delay operations and multiple devices. The computing performance of this system in the second-order nonlinear dynamical task is shown in Supplementary Fig. S13.1c. The results show that the prediction error of the reservoir computing system, evaluated by NMSE, is improved by an order of magnitude by this multiple-FeFET system. The improved prediction capability can also be seen in the better linear relation of the $d(n)$-$y(n)$ plot (reservoir computing system output $y(n)$ closer to the ideal output $d(n)$) shown in Supplementary Fig. S13.1d as compared to the reservoir computing by a single FeFET in Supplementary Fig. S12.1c.

Note that this is an example for a simple demonstration, in which there is no nonlinear interaction between each parallel-connected FeFETs and thus the nonlinearity of the physical reservoir is not improved by this integration. More sophisticated integration systems including the interaction between FeFETs can be considered depending on the properties of reservoir (namely short-term memory and nonlinearity) required by given tasks.

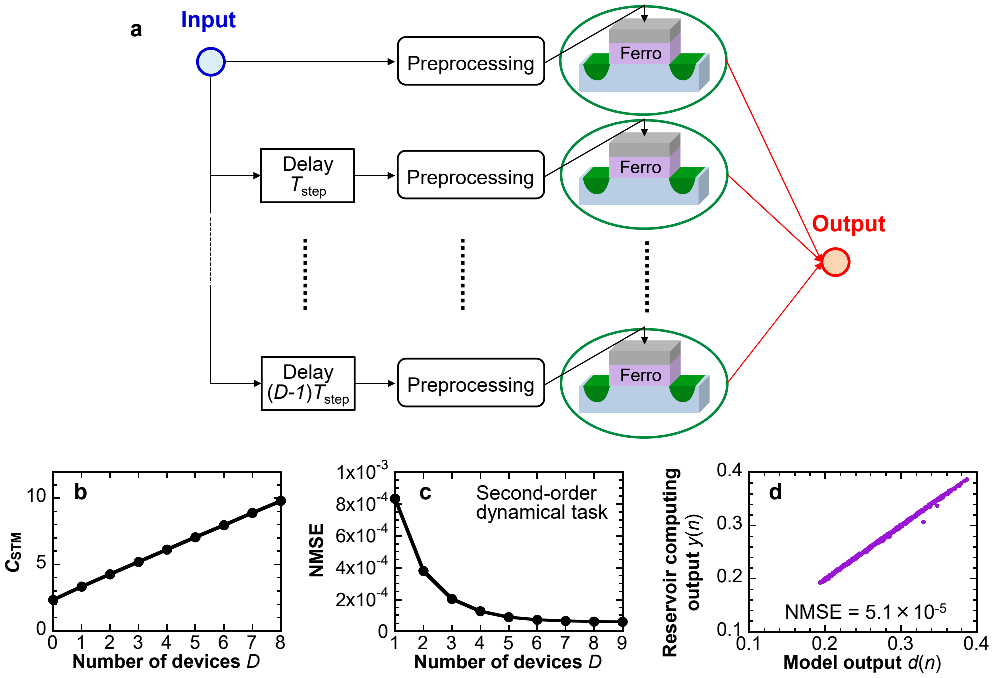

Supplementary Fig. S13.1 | Example of a reservoir computing system with multiple FeFETs. a, Schematic of the reservoir computing system with D FeFET devices and delay operations. The reservoir states $\mathbf{x}(n)$ for a single FeFET were experimentally obtained similarly to the reservoir computing introduced so far in this work, and the parallel connection was carried out through simulation. **b,** Short-term memory capacity C_{STM} of D-parallel systems. **c-d,** Capability of D-parallel system to predict the output of the second-order nonlinear dynamical system, evaluated by NMSE (**c**) and $y(n)$-$d(n)$ plot (**d**). NMSE as small as 5.1×10^{-5} is obtained from the 9-device parallel system. Much improved prediction capability can be observed from the linearity of $y(n)$-$d(n)$ plot as compared to single FeFET.

Supplementary references

1. Toprasertpong, K., Tahara, K., Takenaka, M. & Takagi, S. Evaluation of polarization characteristics in metal/ferroelectric/semiconductor capacitors and ferroelectric field-effect transistors. *Appl. Phys. Lett.* **116,** 242903 (2020).
2. Bartic, A. T., Wouters, D. J., Maes, H. E., Rickes, J. T. & Waser, R. M. Preisach model for the simulation of ferroelectric capacitors *J. Appl. Phys.* **89**, 3420–3425 (2001).
3. Mulaosmanovic, H., Mikolajick, T. & Slesazeck, S. Accumulative polarization reversal in nanoscale ferroelectric transistors. *ACS Appl. Mater. Interfaces* **10,** 23997–24002 (2018).
4. Toprasertpong, K., Takenaka, M. & Takagi, S. Direct observation of charge dynamics in FeFET by quasi-static split C-V and hall techniques: Revealing FeFET operation. In *Proc. 2019 IEEE International Electron Devices Meeting* 570–573 (IEEE, 2019).
