# Supplementary material for: Reservoir computing on a silicon platform with a ferroelectric field-effect transistor
Source: Commun Eng. 2022 Aug 5;1:21. doi: 10.1038/s44172-022-00021-8 (PMC10956125; doi:10.1038/s44172-022-00021-8)
Supplement: Supplementary file 1 — Toprasertpong_PR File [file 44172_2022_21_MOESM1_ESM.pdf]

nature portfolio

Peer Review File

Reservoir computing on silicon platform with a ferroelectric field-effect transistor

Open Access This file is licensed under a Creative Commons Attribution 4.0 International License, which permits use, sharing, adaptation, distribution and reproduction in any medium or format, as long as you give appropriate credit to the original author(s) and the source, provide a link to the Creative Commons license, and indicate if changes were made. In the cases where the authors are anonymous, such as is the case for the reports of anonymous peer reviewers, author attribution should be to 'Anonymous Referee' followed by a clear attribution to the source work. The images or other third party material in this file are included in the article's Creative Commons license, unless indicated otherwise in a credit line to the material. If material is not included in the article's Creative Commons license and your intended use is not permitted by statutory regulation or exceeds the permitted use, you will need to obtain permission directly from the copyright holder. To view a copy of this license, visit http://creativecommons.org/licenses/by/4.0/.

Reviewers' comments:

Reviewer #1 (Remarks to the Author):

In their manuscript, “Reservoir computing on silicon platform with a ferroelectric field-effect transistor”, Toprasertpong et al. demonstrarte a time-multiplexed reservoir based on a ferro-electric field effect transistor (FeFET). Using the well-established temporal embedding technique, they define virtual nodes which enable the expansion of the single-node reservoir’s dimensionality by the temporal-multiplexing factor. Other than in the usual delay RC, here the temporal nature of the process comes from device-internal nonlinear dynamics which induce the temporal aspect in the nonlinear transformations due to percolation and other processes within the Hafnium layer of the FeFET. The manuscript is well written, the presented data is sound and the manuscript combined with its supplementary material contains a substantial body of results, characterization and analysis.

Leveraging device-internal effects for inducing the short-term memory is interesting in general. However, the manuscript does not significantly advance beyond the state of the art. The virtual embedding of the reservoir state means that the current concept most likely has no technological relevance, as auxiliary infrastructure providing temporal multiplexing and demultiplexing will substantially exceed the complexity of non-temporally multiplexed systems. The proposed device is novel, but it does not fundamentally advance the field compared to other electronic components that demonstrated the identical concept before. The field has substantially advanced in the recent past, and more novelty, more complete systems and demonstration of more demanding benchmark tests are now required for high-impact publication. CMOS compatibility of a single component is not sufficient, and indeed was already demonstrated. While the manuscript is of high quality, I cannot recommend publication in Nature Communications Engineering due to the lack of novelty and potential impact.

Reviewer #2 (Remarks to the Author):

FeFET devices are proposed as reservoir in a system for time series data processing. A single FeFET reservoir is demonstrated and its performance assessed in two scenarios. The study is thorough and the results are solid

The Authors already published a manuscript on IEEE Symposium on VLSI Technology 2020 (ref. 20) dealing with similar topics. Is the submitted manuscript an extended version of the other manuscript? The aspects of novelty of this manuscript compared to the VLSI must be clearly stated.

If I am not mistaken, Figure 4 and Figure 5 are the same, so it is not possible for me to assess the results that should refer to Figure 4.

In my opinion, the manuscript would benefit from a characterisation of the FeFET, maybe to be added in the supplementary information. Indeed, since the reservoir is made of a single FeFET, its electrical behaviour is of paramount importance for the performance of the proposed system. In particular, it would be worth discussing how the device properties affect the system, in particular the stochastic properties of the device and its variability, and the characteristics of the short term memory, e.g., possible electrical tunability and achievable time window.

Typos and minor points:
- In Page 2, to my understanding, when Supplementary Figure S1 is referred to, it should be Figure S1(a), whereas Figure S2 should be Figure S1(b).
- Page 3, line 57: "destructive" sounds to me a bit negative. Maybe the Authors should consider to use "disruptive" instead, which has a more positive connotation.

- The difference between the internal states of the FeFET reservoir in Section 7 of the supplementary information is not very clear. As also the Authors noticed, the difference is small. Would it be possible for the Authors to directly plot the difference between the states rather than showing them overlapped as it is now in supplementary Figure S9?
- In Section 9 of the supplementary information, the Authors write "Both the W_res and W_in are not updated by training, but are reconstructed in each run of simulation." This sentence is not very clear, it seems like the Authors tuned the weights to get the result they wanted, which I am sure it is not the case. Is it possible to re-write the sentence?

Reviewer #3 (Remarks to the Author):

The authors presents a reservoir computing system using a FeFET transistor by exploiting its polarization dynamics. Overall, the work is quite timely and interesting and I think it would be of interest to the research community.

The only recommendation I have is to clarify how the fabricated devices are mapped over to generate the results in the RC tasks shown in Fig. 4. Are the dynamics extracted from a sample of devices, and then computationally modelled via Eq (1)? Please clarify what component of the study was done on hardware and what was performed computationally.

Reply to reviewer comments for Manuscript COMMSENG-22-0001 "Reservoir computing on silicon platform with a ferroelectric field-effect transistor"

Kasidit Toprasertpong

The authors would like to thank the editor and the reviewers for assisting in making this manuscript a more complete and succinct document. In our revision, we took into account all specific and technical comments by the reviewers as detailed below. In this report, red texts correspond to the revised/added texts in the manuscript, gray texts correspond to the original texts in the manuscript referred here for reference, and brown texts correspond to the reviewers' comments.

Reviewer #1

In their manuscript, "Reservoir computing on silicon platform with a ferroelectric field-effect transistor", Toprasertpong et al. demonstrarte a time-multiplexed reservoir based on a ferro-electric field effect transistor (FeFET). Using the well-established temporal embedding technique, they define virtual nodes which enable the expansion of the single-node reservoir's dimensionality by the temporal-multiplexing factor. Other than in the usual delay RC, here the temporal nature of the process comes from device-internal nonlinear dynamics which induce the temporal aspect in the nonlinear transformations due to percolation and other processes within the Hafnium layer of the FeFET. The manuscript is well written, the presented data is sound and the manuscript combined with its supplementary material contains a substantial body of results, characterization and analysis.

1. *Leveraging device-internal effects for inducing the short-term memory is interesting in general. However, the manuscript does not significantly advance beyond the state of the art. The virtual embedding of the reservoir state means that the current concept most likely has no technological relevance, as auxiliary infrastructure providing temporal multiplexing and demultiplexing will substantially exceed the complexity of non-temporally multiplexed systems. The proposed device is novel, but it does not fundamentally advance the field compared to other electronic components that demonstrated the identical concept before. The field has substantially advanced in the recent past, and more novelty, more complete systems and demonstration of more demanding benchmark tests are now required for high-impact publication. CMOS compatibility of a single component is not sufficient, and indeed was already demonstrated. While the manuscript is of high quality, I cannot recommend publication in Nature Communications Engineering due to the lack of novelty and potential impact.*

   We would like to address that even though this manuscript demonstrates reservoir computing focusing on the properties of a single FeFET and the use of an existing concept of virtual nodes, the reservoir computing technology using an FeFET has many unique features that provide new technical aspects and contributes to the progress of research fields. We would like to explain the technological impacts of our proposal in the following aspects.

- **Technological aspect on reservoir computing**

~ Comparison with other CMOS-compatible devices ~

We would like to address the technological benefits of our proposed FeFET reservoir over existing physical reservoirs. First, we would like to limit our discussion to CMOS-compatible reservoirs since our ultimate goal of this proposal is to implement reservoir computing hardware on CMOS systems.

One notable feature of FeFETs is that they are *a single device with a multi-terminal structure*. Unlike other CMOS-compatible technologies considered for reservoir computing such as two-terminal memristors, FeFETs basically have four terminals, namely gate, drain, source, and substrate (body). In conventional two-terminal devices, only one waveform of voltage $V(t)$ is applied and one waveform of current $I(t)$ or resistance $R(t)$ is extracted. On the other hand, in four-terminal devices, there are various possible approaches to control the reservoir and several possible ways to utilize the dynamics to perform reservoir computing.

- *Spatial intermixing*: As shown in **Fig. 5b-c** and explained in **page 9 line 20**, we can extract the currents from the three terminals of an FeFET to form reservoir-state vectors. The capability to extract dynamics from different spatially-separated terminals allows us to *effectively utilize the spatial dynamics of polarizations and charges in FeFETs* (**Supplementary Fig. S4.1**). As the current from each terminal corresponds to different dynamics in FeFET (**Supplementary Fig. S5.1**), utilizing all the three currents increases the dimensionality of the reservoir state vectors and significantly improves the reservoir computing performance. This technique using signals from multiple terminals is *a unique feature of four-terminal FeFETs that cannot be implemented in typical two-terminal devices*. In two-terminal devices, even though there might be both temporal and spatial dynamics inside the devices, only limited information of the dynamics can be extracted from the single readout terminal.
- *Design flexibility*: We have more flexibility on the system design when the reservoir part has multiple terminals. For example, we can employ a system that extracts all the currents for better computing performance, or one that extracts only some currents when we need systems to be simple. More choices of possible reservoir designs lead to better tunability of the reservoir computing system for given applications.

  Furthermore, the multi-terminal structure of FeFETs also has a benefit in the fine-tuning capability of the reservoir properties by applying a bias voltage to their remaining terminals. As shown in **Supplementary Section 11** and explained in **page 10 line 2**, applying a bias voltage to the substrate while applying the input signal to the gate of an FeFET allows us to adjust the reservoir properties. The optimum substrate bias that gives the best performance is different for the temporal-XOR, parity-check, and second-order nonlinear dynamical tasks. Because the required reservoir properties can be different depending on the complexity of the computational tasks, the optimum reservoir

conditions can also be dependent on tasks. Considering that in actual applications, hardware (devices) cannot be reconstructed after it is physically integrated into a chip, the capability of fine-tuning by simply applying bias would make FeFET reservoirs highly promising for the implementation as reservoir computing hardware. Note that the fine-tuning capability in **Supplementary Section 11** is only one demonstration for the proof of concept, which can be improved by further optimization, e.g., by applying biases on the drain and source terminals. We hope that our demonstration will bring the researcher's attention to tunable physical reservoirs, which the authors believe to be essential for the hardware implementation of the reservoir computing technology.

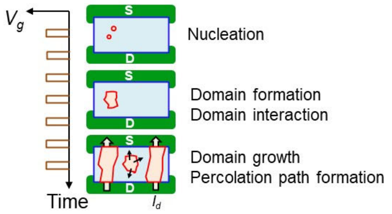

Supplementary Fig. S4.1 | Top-view schematic of polarization dynamics in an FeFET. Under applied gate voltage V_g, electric field drives a variety of dynamics inside the ferroelectric insulator. This includes domain nucleation and formation, which is known to have an accumulative behavior (domain formation occurs only after accumulated input is over a certain voltage/time limit[3]), interaction between domains, and domain growth. The channel current flows only through the percolation paths which are distributed according to the polarization distribution and the polarization/charge interaction. The different time scales of each dynamic results in nonlinear response to the input.

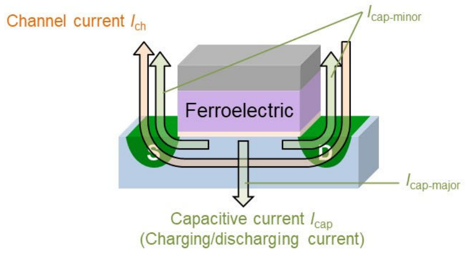

Supplementary Fig. S5.1 | Main components of current detected from the drain, source, and substrate of an FeFET. The channel current I_{ch}, driven by the drain voltage, and the capacitive current I_{cap}, driven by the time-derivative of gate voltage, are the main current components in an FeFET. In an inversion-mode FeFET, the capacitive current flows through the substrate ($I_{cap\text{-}major}$) when the flowing charges are the majority carrier and through the source/drain when the flowing charges are the minority carriers ($I_{cap\text{-}minor}$).

page 9, line 20: The performance of the reservoir computing system using multi-terminal FeFETs is compared with the results when only the drain current $I_d(t)$ is used in reservoir computing. Figs. 5a,d show that utilizing currents from multiple terminals results in better computing performance. These current components are determined by different physical mechanisms in FeFETs (see Supplementary Section 5) and are equivalent to different nonlinear transformations. Hence, constructing the reservoir-state vectors by combining the $I_d(t)$, $I_s(t)$, and $I_{sub}(t)$ waveforms improves the dimensionality of the nonlinear transformation performed by the reservoir and results in higher reservoir computing performance.

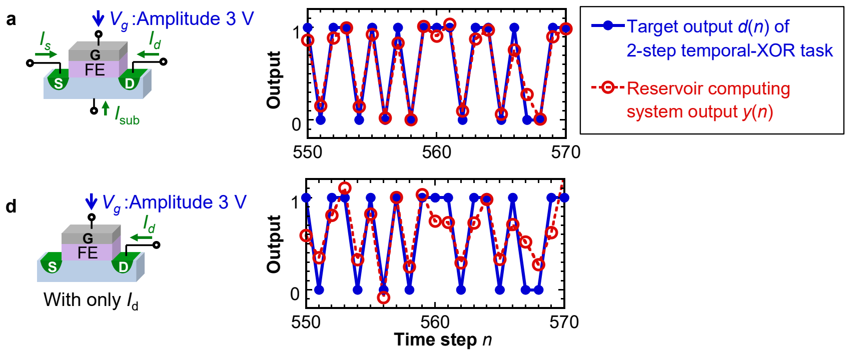

Fig. 5 | Comparison of reservoir computing system output for the 2-step temporal-XOR task under alternative operation schemes. a-b, FeFETs under an input voltage amplitude of 3 V employed in this study (**a**) **…. d,** Reservoir system in which only drain current of FeFET is employed in reservoir computing. The reservoir employing multiple current components consisting of drain current, source current, and substrate current of FeFETs (**a**) exhibits higher computing performance than that using only one single current component (**d**). {(b) and (c) are omitted here since they are for different discussions}

page 10, line 2: Moreover, we can change the bias voltage on the remaining terminals to further adjust the dynamics inside FeFETs for more efficient computing of given tasks, as demonstrated in Supplementary Section 11. Therefore, the multi-terminal structure of FeFETs is a remarkable feature that contributes to high design flexibility of FeFET-based reservoir computing systems.

Supplementary Section 11: **Reservoir tunability by substrate bias**

As a demonstration of the design flexibility of reservoir computing using an FeFET with multiple terminals, we examine the reservoir computing performance under different substrate biases. Supplementary Fig. S11.1 shows C_{XOR} from the temporal-XOR task, C_{PC} from the parity-check task, and NMSE results from the second-order nonlinear dynamical task under the substrate biases varied from 0 V to -5 V. We can see that substrate biases that give the maximum performance is task-dependent: -2.25 V for the temporal-XOR, 0 V for the parity-check task, and -5 V for the second-order nonlinear dynamical tasks. The difference in optimum points is due to the fact that different tasks have different computational complexity and may require different properties of the reservoir to solve. By applying the FeFET substrate bias, the depletion region in the semiconductor channel is modulated, affecting the dynamics of the drain current, source current, and substrate current. This indicates that the multi-terminal feature of FeFETs enables fine-tuning of the FeFET reservoir to handle different tasks effectively with the same devices and hardware configurations. This feature would be beneficial in practical implementation in which the hardware cannot be reconstructed after it is physically integrated into a chip.

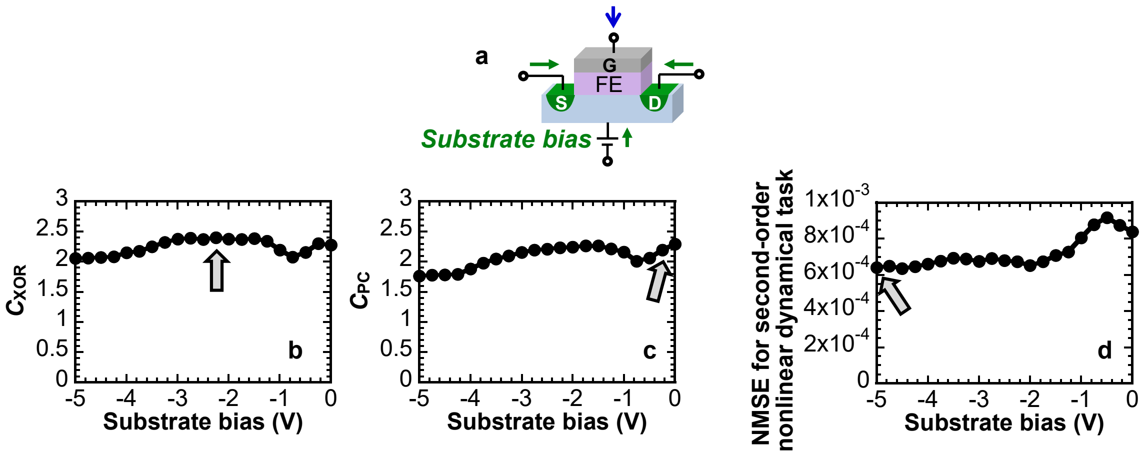

Supplementary Fig. S11.1 | Tunable reservoir properties of an FeFET by substrate bias. a, Operation schematic. **b,** Temporal-XOR capacity C_{XOR}. **c,** Parity-check capacity C_{PC}. **c,** NMSE for second-order nonlinear dynamical task. Note that high C_{XOR}, high C_{PC}, and low NMSE correspond to higher performance.Within the -5 V ≤ substrate bias ≤ 0 V range with a 0.25 V measurement step, the optimal substrate biases are -2.25 V, 0 V, and -5 V for the temporal-XOR, parity-check, and second-order nonlinear dynamical tasks, respectively, as indicated by the arrows in the figures.

- ## Technological aspect on reservoir computing (Cont.)

~ Comparison with CMOS circuits ~

We would like to compare with previous demonstrations of reservoir computing using nonlinear CMOS circuits composed of nonlinear electronic components. The circuit-based reservoir hardware is a type of CMOS-compatible technology, but it should be noted that there is a large difference in the application aspect. A circuit-based reservoir computing hardware can be designed with a number of active nonlinear electronic components such as op-amps to realize the reservoir function with high nonlinearity, dimensionality and/or short-term memory. However, this usually comes with significant area and power-consumption penalties: the more functional the circuit, the more area and power it tends to consume. The high power consumption is considered challenging for circuit-based reservoirs, considering that the main goal of physical reservoir computing is to realize computing-efficient hardware to replace software-based reservoir computing systems. The large area might not be an issue for applications requiring a single reservoir, but the integration of multiple reservoirs could be required in actual LSI (large-scale integration) implementation, in which the area would be a critical factor.

On the other hand, our reservoir computing systems using FeFET are considered promising for the low-power, scaled reservoir computing hardware. It can be functionalized even only by a single FeFET. Even though our device in this demonstration has a macro-meter size due to our university's facilities, the size can be scaled down to the order of ten nanometers realized when fabricated in advanced CMOS technology nodes. Furthermore, using nonlinear physical mechanisms such as polarization-polarization and polarization-charge interactions instead of highly-active electrical components allow FeFET reservoirs to operate with low power consumption for efficient reservoir computing operation.

~ Comparison with CMOS-non-compatible devices ~

Even though the reviewer's concern might be about the comparison among CMOS-compatible reservoirs, we would like to make a short comment on reservoirs that are not compatible with CMOS technologies. There are already a lot of proposed physical reservoirs, but we should note that the application of each reservoir system depends on its nature. For example, mechanical reservoirs would be promising for robotic applications. Since our goal is to implement the reservoir computing hardware on a silicon chip together with existing CMOS technologies, the reservoir must be compatible with current CMOS technologies, and FeFET is a technology that well satisfies this requirement.

- **Flexible physical reservoir for fundamental research on physical reservoirs**

The high flexibility of reservoir computing using FeFETs at the material level (not shown in this study), the device level, and the circuit level helps fundamental research for the physical reservoir computing community, in which the understanding of the relation between the physical dynamics and reservoir computing performance is still needed. The flexible design of FeFET-based reservoir computing systems to control their nonlinear dynamic properties would enable FeFET to be a potential tool for systematic studies of reservoir computing and promotes a deep understanding of the relation between the dynamics of physical systems and the reservoir computing performance.

- **Contribution on the physical reservoir community in an implementation aspect**

We consider FeFETs promising for the reservoir computing application not only because FeFETs can perform reservoir computing with satisfactory performance and have high design flexibility as previously discussed, but also because reservoir computing using FeFETs has a variety of possible options for integration in existing CMOS technologies. As discussed in **page 11 line 19**, FeFETs can be implemented either in front-end-of-line close to logic devices for efficient computing, in back-end-of-line integrated together with other emerging memory technologies, or in 3D architectures for high-density stand-alone reservoir computing. This implies that FeFET-based reservoir computing systems can be implemented in a wide range of architectures depending on the system design. This feature is different from other CMOS-compatible devices or hardware by which the integration architecture might be limited.

Furthermore, as discussed in **page 12 line 12**, FeFETs have also been investigated as promising logic devices, memory devices, AI-accelerator devices, and peripheral devices. For this reason, reservoir computing using FeFETs can be easily implemented together with other FeFETs. We may consider an "FeFET-based intelligent chip" which is mainly composed of functional FeFETs as it would be possible to be fabricated together in the same process line. This is one scenario that we can expect from reservoir computing using FeFETs but is not easily achievable by other technologies.

The FeFET technology is currently in a stage close to practical implementation as seen from the fact that the FeFET technology is now made available in many leading semiconductor foundries. This rapid development of the FeFET technology supports the feasibility of the implementation of FeFET-based

reservoir computing hardware as well as the integration with other FeFET-based functions and existing CMOS technologies in practical LSI systems.

page 11, line 19: Furthermore, a variety of design for further extension of FeFET reservoir computing systems is feasible as HfO_2-based FeFETs can be implemented by several approaches ranging from front-end-of-line[24], back-end-of-line[46] to 3D architectures[47].

page 12, line 12: FeFET-based reservoir computing systems, thanks to their CMOS compatibility and scalability, make it feasible to implement the reservoir computing function on a silicon chip with existing CMOS technologies as well as with FeFETs being employed in logic, memory, and computing-in-memory towards the next-generation intelligent systems.

- **Contribution to the FeFET community**

  FeFETs are devices that have been intensively studied in recent years in many aspects ranging from logic, memory, computing-in-memory, and peripheral-circuit applications. Most applications of FeFETs so far utilize only the static properties of ferroelectric polarization, such as nonvolatile UP and DOWN polarization states. Even though there are a lot of studies reporting complex nonlinear dynamics or ferroelectric polarization, which becomes more complicated when the ferroelectric is in contact with semiconductor in FeFETs, FeFETs so far could not easily find applications that can functionalize their complex dynamic properties. Our proposed FeFET-based reservoir computing utilizing the dynamics properties of FeFETs would be an exciting demonstration that provides new aspects of FeFET applications and triggers the FeFET community to explore new possibilities of FeFETs by further exploiting the FeFETs' dynamic properties and/or other unexplored characteristics of FeFETs.

  This discussion has been made in **page 3 line 23**.

  page 3, line 23: HfO_2-based FeFETs have been investigated in a wide range of applications including logic[25], nonvolatile memory array[24], ternary content-addressable memory[26], and computing-in-memory[27-29], most of which rely on the static properties of the ferroelectric polarization. The use of FeFETs for reservoir computing extracts the unexplored potential of their rich dynamics including the domain dynamics and the coupling dynamics between polarizations and charges for AI computing, opening up an alternative application of FeFETs.

Even though our manuscript focuses on the demonstration and analysis of a single FeFET for reservoir computing application, we believe that the proposed reservoir computing concept using an FeFET brings a lot of new possibilities to both the physical reservoir computing field and the FeFET field. It will advance the fields both in fundamental understanding for a scientific purpose and providing hardware with high feasibility for practical applications, and would be of interest to the readers of *Communications Engineering*.

==

Reviewer #2

FeFET devices are proposed as reservoir in a system for time series data processing. A single FeFET reservoir is demonstrated and its performance assessed in two scenarios. The study is thorough and the results are solid

1. *The Authors already published a manuscript on IEEE Symposium on VLSI Technology 2020 (ref. 20) dealing with similar topics. Is the submitted manuscript an extended version of the other manuscript? The aspects of novelty of this manuscript compared to the VLSI must be clearly stated.*

This manuscript is a full paper that has been extended from the preliminary results published in the conference named IEEE Symposium on VLSI Technology 2020 (ref. 20). We would like to point out that we have added several new contents and discussions beyond the content in the conference paper. In the conference paper, we proposed that an FeFET can be employed in reservoir computing by applying the gate voltage V_g as the input of reservoir and sampling the drain current I_d of FeFET to form a reservoir state. The discussion has been limited to the points that our technology can be used for real-time machine learning of time-series data. The original points of this manuscript compared to the conference paper are listed below.

(1) *Multiple terminal FeFET*: While we have utilized only the drain current I_d in our conference paper, in this study we have proposed the utilization of all the terminals of FeFETs, namely the drain current I_d, the source current I_s, and the substrate current I_{sub}, to form the reservoir states in reservoir computing. This approach helps us extract the nonlinear dynamics of FeFETs that cannot be easily extracted only from I_d, and hence improves the dimensionality of the reservoir state. This is also a demonstration of how to exploit the notable multi-terminal feature of FeFET, which is an advantage over other reservoir computing technologies. In the pre-reviewed version of the manuscript, as pointed out by the reviewer, we have not clearly stated the difference from the previous conference paper and provided insufficient discussion. We have clarified this point and extended the discussion in **page 6 line 2** and **page 9 line 20** in the revised manuscript. In addition, we demonstrate that the multi-terminal structure of FeFETs makes FeFET-based reservoir computing systems flexible to be fine-tuned according to target tasks. This will be discussed in the reply of question 3.

(2) *Second-order nonlinear dynamical task and Temporal-XOR*: The previous conference paper has shown only the computing performance under the delay task and the parity-check task. In this study, we have demonstrated the computing performance under the second-order nonlinear dynamical task aiming to predict unknown time-series data, which is a task closer to practical applications of reservoir computing. Moreover, we have added the temporal-XOR task for completeness, considering that the nonlinearity of the temporal-XOR task is between the delay task and the parity-check task. (c.f. $d(n)=u(n-\tau)$ for the delay task, $d(n)=u(n)\oplus u(n-\tau)$ for the temporal-XOR task, and $d(n)=u(n)\oplus u(n-1)\oplus ...\oplus u(n-\tau)$ for the parity-check task) We have put the reference number to the

tasks that we have tested in our previous works (but with different results as only I_d was used) in **page 7 line 17** and **page 8 line 12**.

(3) *Comparison with software-based reservoir computing*: We have additionally made a comparison between our FeFET-based reservoir computing hardware with reservoir computing software as a reference for the readers who might also be interested in software reservoir computing, as described in **page 8 line 23** and in **Supplementary Section 9**

(4) *More details on the experiments and methods*: There is only limited explanation on the experimental method in the 2-page conference paper. In this manuscript, we have added several discussions on the experiments and methods so that the readers can understand the actual experiments and reproduce the results if needed, including

   a. Actual waveforms of currents used in the reservoir computing (**Fig. 2** and **Supplementary Section 7**)
   b. Details of equations using in the calculation (Eq. (2)-(10))
   c. Method to determine the ridge parameter in the ridge regression and description on how it decreases the noise of the calculated weight matrix **(Supplementary Section 8**)
   d. Note on the nonideality of the experimental setup that may lead to incorrect interpretation if careful intention is not paid (**Supplementary Section 6 and 12**)

(5) *Use of knn method to analyze the t-SNE results*: We employed the *k*NN method to evaluate the results of t-SNE analysis and quantitatively discuss the capability of high-dimensional transform of the FeFET in **page 7 line 3**. In the conference paper, only the t-SNE plots are shown with only qualitative discussion.

(6) *Discussion on possible origins of nonlinear transform by FeFETs*: While the conference paper mainly provides the experimental results of computing performance, we made an extensive discussion in this manuscript about the nonlinear physical mechanisms in FeFET that contribute to the reservoir computing performance, such as hysteretic properties, polarization nucleation, domain formation, polarization-polarization interaction, polarization-carrier interaction, and superposition of channel current and capacitive current. This is discussed in **page 5 line 3** and in **Supplementary Section 3-5**

(7) *Discussion on the future outlook on multiple-device integration and system implementation*: In the conference paper, we have focused only on a reservoir computing system with a single FeFET. This manuscript discussed the possible future outlook of this FeFET reservoir computing technology. Integration using multiple FeFETs would improve the performance of FeFET reservoir computing as demonstrated in **Supplementary Section 13**. We have also discussed in **page 12 line 12** that the FeFET reservoir computing is feasible to be integrated with CMOS and FeFETs employed in logic, memory, and computing memory to implement an intelligent system on a silicon chip.

The reviewer can see that even though the concept itself has been proposed in the previous conference paper, most parts of this manuscript are new contents. As it will take too much space to state all the above in this manuscript, we decide to clarify only (1) and (2), which are the main experimental parts.

page 6, line 2: In contrast with the FeFET reservoir system in our previous report[20] in which only $I_d(t)$ was used to form the reservoir state, the reservoir system proposed in this Article utilizes all three current terminals $I_d(t)$, $I_s(t)$, $I_{sub}(t)$ for reservoir computing. Since currents $I_d(t)$, $I_s(t)$, $I_{sub}(t)$ reflect different physical dynamics in the FeFET (see Supplementary Section 5 and ref. [32]), utilizing these current components are expected to increase the dimensionality of the reservoir state needed for computing.

page 9, line 20: The performance of the reservoir computing system using multi-terminal FeFETs is compared with the results when only the drain current $I_d(t)$ is used in reservoir computing. Figs. 5a,d show that utilizing currents from multiple terminals results in better computing performance. These current components are determined by different physical mechanisms in FeFETs (see Supplementary Section 5) and are equivalent to different nonlinear transformations. Hence, constructing the reservoir-state vectors by combining the $I_d(t)$, $I_s(t)$, and $I_{sub}(t)$ waveforms improves the dimensionality of the nonlinear transformation performed by the reservoir and results in higher reservoir computing performance.

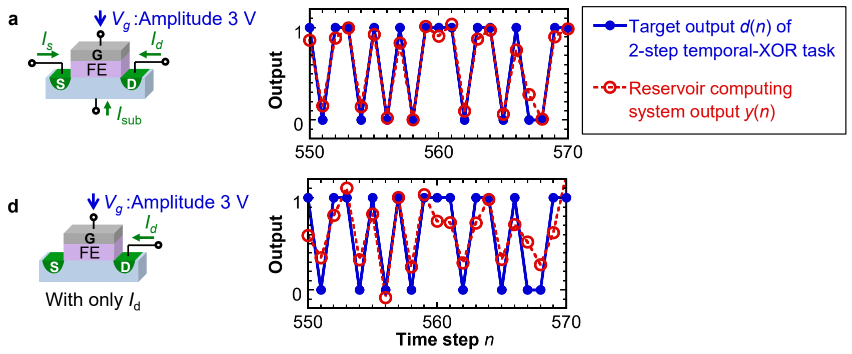

Fig. 5 | Comparison of reservoir computing system output for the 2-step temporal-XOR task under alternative operation schemes. a-b, FeFETs under an input voltage amplitude of 3 V employed in this study (**a**) **…. d,** Reservoir system in which only drain current of FeFET is employed in reservoir computing. The reservoir employing multiple current components consisting of drain current, source current, and substrate current of FeFETs (**a**) exhibits higher computing performance than that using only one single current component (**d**). {(b) and (c) are omitted here since they are for different discussions}

page 7, line 17: First, delay tasks were carried out to evaluate the short-term memory capacity (C_{STM}), corresponding to the short-term memory characteristics[20,40].

page 8, line 12: We investigate the computing capability of nonlinear tasks by examining the temporal-XOR task[17] and the parity-check task[20,41].

2. *If I am not mistaken, Figure 4 and Figure 5 are the same, so it is not possible for me to assess the results that should refer to Figure 4.*

We apologize for the issue. **Fig. 4** was mistakenly replaced by **Fig. 5** in our initial submission, but we have gone through the editorial system to replace our correct file with the editor’s permission. I am not sure

about the reason but you might accidentally have the access to the previous version. This revised manuscript should show the correct figure.

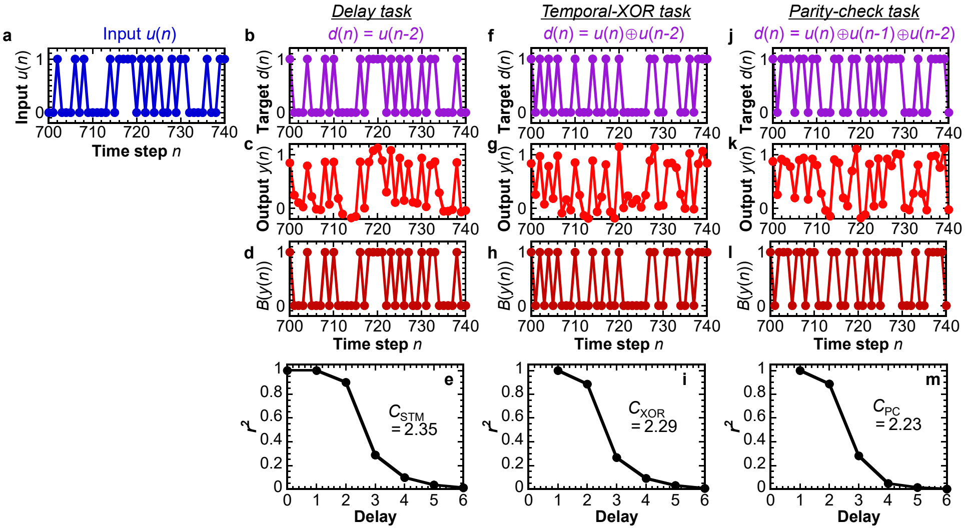

Fig. 4 | Reservoir computing results for delay, temporal-XOR, and parity-check tasks. a, Input signal $u(n)$ of the test data. Only 40 time steps are shown here. **b-d,** Target output $d(n) = u(n\text{-}2)$ (**b**), computed system output $y(n)$ (**c**), and binarized output $B(y(n))$ (**d**) of the trained FeFET reservoir system for the 2-step delay task. Here, $B(y(n)) = 0$ when $y(n) < 0.5$ and $B(y(n)) = 1$ when $y(n) \geq 0.5$. **e,** Squared correlation coefficient r^2 between the computed system output $y(n)$ and the target output $d(n) = u(n\text{-}\tau)$ for the τ-step delay tasks. **f-h,** Target signal $d(n) = u(n) \oplus u(n\text{-}2)$ (**f**), computed system output $y(n)$ (**g**) and binarized output $B(y(n))$ (**h**) for the 2-step temporal-XOR task, where $\oplus$ is the XOR operation. **i,** r^2 between $y(n)$ and $d(n) = u(n) \oplus u(n\text{-}\tau)$ for the τ-step temporal-XOR task. **j-l,** Target $d(n) = u(n) \oplus u(n\text{-}1) \oplus u(n\text{-}2)$ (**j**), output $y(n)$ (**k**) and binarized output $B(y(n))$ for the 2-step parity-check task. **m,** r^2 between $y(n)$ and $d(n) = u(n) \oplus \dots \oplus u(n\text{-}\tau) = \sum_{l=0}^{\tau} u(n-l) \pmod 2$ for the τ-step parity-check task. C_{STM}, C_{XOR}, and C_{PC}, which are the sum of r^2 for delay step $\tau \geq 1$, are shown in the corresponding figures.

3. *In my opinion, the manuscript would benefit from a characterisation of the FeFET, maybe to be added in the supplementary information. Indeed, since the reservoir is made of a single FeFET, its electrical behaviour is of paramount importance for the performance of the proposed system. In particular, it would be worth discussing how the device properties affect the system, in particular the stochastic properties of the device and its variability, and the characteristics of the short term memory, e.g., possible electrical tunability and achievable time window.*

We would like to thank the reviewer for the advice on an additional discussion that will help enhance our understanding of the operation of FeFET-based reservoir computing. We have added a discussion in **page 9 line 10** about the dependence on the input voltage amplitude. Since the external voltage can drive the ferroelectric polarization dynamics only when the corresponding electric field is sufficiently large (~coercive field), the input voltage amplitude is a key factor that determines the ferroelectric behavior in FeFETs and investigation of this factor would gain our understanding of the role of ferroelectric in the reservoir computing. The results are shown in **Fig. 5a-b** and **Supplementary Fig. S10.1**. The reservoir computing performance significantly drops when the input gate-voltage amplitude is less than 1.5 V, which is just the same voltage amplitude that the ferroelectric hysteresis in I_d-V_g characteristics disappears. In other words,

the FeFET-based reservoir computing can be performed only when the voltage is sufficient to modulate the ferroelectric polarization. This result confirms that the ferroelectric polarization in FeFETs is the origin of the computing performance in FeFET reservoirs.

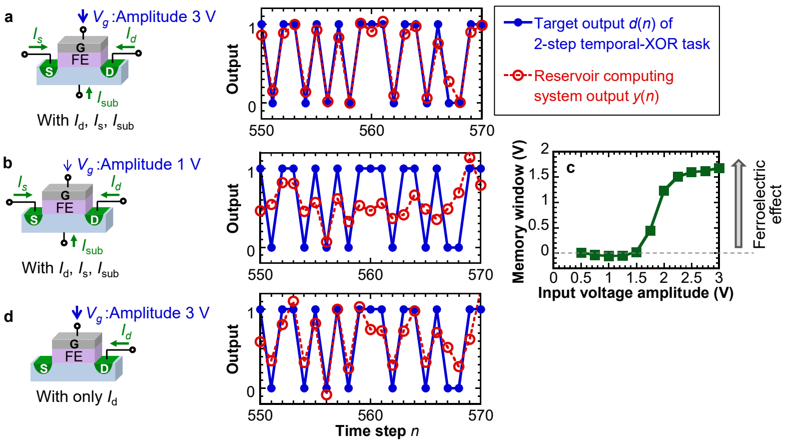

Fig. 5 | Comparison of reservoir computing system output for the 2-step temporal-XOR task under alternative operation schemes. a-b, FeFETs under an input voltage amplitude of 3 V employed in this study (**a**) and under a lower amplitude of 1 V (**b**). **c,** Memory window of FeFETs evaluated from the transfer characteristics. The ferroelectric behavior of the FeFET can be observed only when operated under an input voltage amplitude above 1.5 V, which is in agreement with the better performance of FeFET-based reservoir computing under higher input voltage amplitude. **d,** Reservoir system in which only drain current of FeFET is employed in reservoir computing. The reservoir employing multiple current components consisting of drain current, source current, and substrate current of FeFETs (**a**) exhibits higher computing performance than that using only one single current component (**d**).

page 9, line 10: We examine the impact of the voltage amplitude of the mask function **v**(n), i.e., the maximum voltage V_g applied to the gate. As shown in Figs. 5a-b for the output time-series and in Supplementary Section 10 for the summary of the performance, the performance of FeFET-based reservoir computing systems substantially degrades when the gate voltage amplitude becomes lower than 1.5 V, which is also the minimum voltage amplitude to observe the ferroelectric hysteresis in the FeFET (Fig. 5c). Below this voltage, the electric field in the ferroelectric film is lower than the coercive field required to modulate the polarization states and thus the device operates similarly to a transistor with no ferroelectric functionality. The result implies that the FeFET can operate as a reservoir only when the polarization dynamics are driven by the voltage input, confirming the contribution of ferroelectric polarization to the reservoir computing capability.

Supplementary Section 10: **Impact of input voltage amplitude**

The actual input signal is converted by the mask function to the voltage waveform to be applied to the gate of FeFET as gate voltage V_g during the reservoir computing operation. The masked waveform **v**(n) in this study is a triangular waveform with a voltage amplitude of 3 V and an offset of 0.5 V. To investigate the impact of input voltage, the reservoir computing performance under different input voltage amplitudes (different mask functions) varied from 0.25 V to 3 V is examined as shown in Supplementary Fig. S10.1. The short-term memory capacity C_{STM}, the temporal-XOR capacity C_{XOR}, and the parity-check capacity C_{PC} significantly decrease when the input voltage amplitude is below 1.5 V. As shown in Fig. 5c in the main text that the ferroelectric memory window in FeFETs can be observed when the input voltage amplitude is above 1.5 V, the similar tendency between the

ferroelectric behavior and the reservoir computing performance implies that the reservoir computing capability is attributed to the ferroelectric polarization in FeFETs.

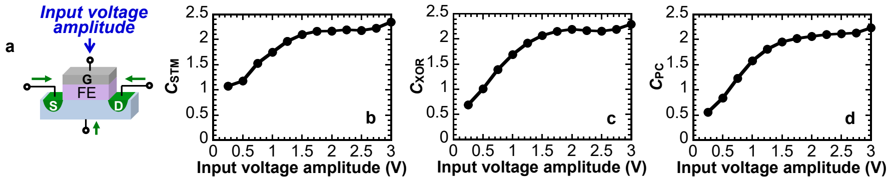

Supplementary Fig. S10.1 | Performance of reservoir computing using an FeFET operated under different input voltage amplitudes. a, Operation schematic. **b,** Short-term memory capacity C_{STM}. **c,** Temporal-XOR capacity C_{XOR}. **d,** Parity-check capacity C_{PC}. All the performances decrease with decreasing input voltage amplitude because of the smaller contribution of the ferroelectric polarization dynamics.

For the effect of stochastic properties, variability, and time window requested by the reviewer, unfortunately, our 5-μm device is too large to study those effects. We need devices with a size of the order of 10 to 100 nm to systematically study those effects. Since it is difficult for our current facility to fabricate new FeFETs with such a small size, we cannot conduct the suggested experiments in this Article. Nevertheless, we agree with the reviewer that such characterization would be essential to understanding the operating mechanism of FeFET-based reservoir computing. We would like to keep this valuable suggestion for our future works when we become capable of evaluating such nano-scale devices.

For the electrical tunability, we have added a simple demonstration in **page 10 line 2** and **Supplementary Section 11**. Taking advantage of the FeFET's multi-terminal structure, FeFET-based reservoir computing can be fine-tuned to different computational tasks by applying bias to the remaining terminals, for instance a substrate bias. Since the required properties of the reservoir can be different in different computational tasks, the capability to fine-tune the reservoir properties only by bias voltage is an interesting feature particularly for reservoir computing hardware, where the hardware configuration is not easily changed after fabrication. We think that the depletion region modulation by substrate bias induces changes in dynamics of current flowing in FeFETs and affects the nonlinearity of the FeFET reservoir.

page 10, line 2: Moreover, we can change the bias voltage on the remaining terminals to further adjust the dynamics inside FeFETs for more efficient computing of given tasks, as demonstrated in Supplementary Section X. Therefore, the multi-terminal structure of FeFETs is a remarkable feature that contributes to high design flexibility of FeFET-based reservoir computing systems.

4. *In Page 2, to my understanding, when Supplementary Figure S1 is referred to, it should be Figure S1(a), whereas Figure S2 should be Figure S1(b).*

Thank you for pointing out our mistake. The reviewer is right that they should be Fig. S1(a) and S1(b) that are referred to. Actually, we have changed the figure numbering to make the readers easy to follow the

figures in Supplementary from Fig. S1, S2, S3, … to S1.1, S2.2, S2.3 corresponding to the section number. In this way, we have corrected to Fig. S1.1(a) and Fig. S1.1(b).

page 2 line 16: A representative model of software-based reservoir computing is echo state networks (ESNs; see Supplementary Fig. S1.1(a)), …

page 2 line 23: … (called *physical reservoir*; see Supplementary Fig. S1.1(b))

5. *Page 3, line 57: "destructive" sounds to me a bit negative. Maybe the Authors should consider to use "disruptive" instead, which has a more positive connotation.*

We would like to thank you for the suggestion. We agree with the reviewer and replaced the word as suggested.

page 3, line 8: … physical reservoir computing has a high potential as a disruptive hardware technology in next-generation edge-AI computing.

6. *The difference between the internal states of the FeFET reservoir in Section 7 of the supplementary information is not very clear. As also the Authors noticed, the difference is small. Would it be possible for the Authors to directly plot the difference between the states rather than showing them overlapped as it is now in supplementary Figure S9?*

Thank you for suggesting out this important point. We have added the plot of the difference in **Supplementary Fig. S7.1(b)** (previous Fig. S9).

Supplementary Fig. S7.1:

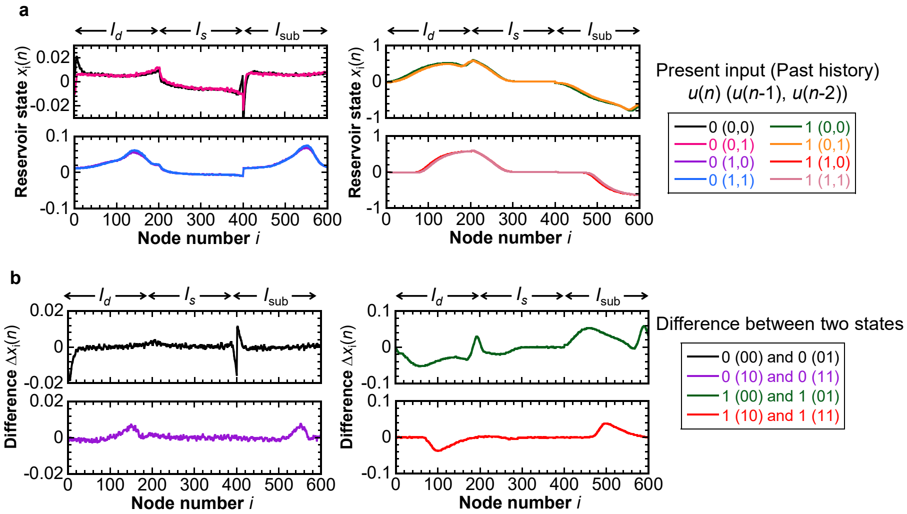

Supplementary Fig. S7.1 | Close comparison of internal states of FeFET reservoir. a, Reservoir states $\mathbf{x}(n)$ corresponding to the same $u(n)$ and $u(n\text{-}1)$ but different $u(n\text{-}2)$ are shown in the same plot. **b,** Difference $\Delta\mathbf{x}(n)$ of the states with the same $u(n)$ and $u(n\text{-}1)$. Even though the shape may look similar, small difference can be observed, which can be clearly distinguished by t-SNE (Fig. 3c) as well as by the readout of reservoir computing (Fig. 4c).

7. *In Section 9 of the supplementary information, the Authors write "Both the W_res and W_in are not updated by training, but are reconstructed in each run of simulation." This sentence is not very clear, it seems like the Authors tuned the weights to get the result they wanted, which I am sure it is not the case. Is it possible to re-write the sentence?*

Thank you for pointing out the ambiguous point. The input weight W_{in} and the reservoir weight W_{res} were randomly determined and we did not intentionally tune them. We have clarified that in **page 11 line 11 of supplementary**.

page 11, line 11 of supplementary: $\mathbf{W}_{\mathbf{res}}$ and $\mathbf{W}_{\mathbf{in}}$ are randomly determined by the mentioned distributions and are fixed (not updated by training) in each run of simulation.

==

Reviewer #3

The authors presents a reservoir computing system using a FeFET transistor by exploiting its polarization dynamics. Overall, the work is quite timely and interesting and I think it would be of interest to the research community.

1. *The only recommendation I have is to clarify how the fabricated devices are mapped over to generate the results in the RC tasks shown in Fig. 4. Are the dynamics extracted from a sample of devices, and then computationally modelled via Eq (1)? Please clarify what component of the study was done on hardware and what was performed computationally.*

We would like to thank the reviewer for pointing out the unclear part. As a proof of concept in this study, the generation of the input sequence (applying V_g) and the mapping of the input to the reservoir state (measuring I_d, I_s, I_{sub}) were carried out on hardware, while the matrix computation in equations (2)-(5) were carried out on software to estimate the actual performance. We have modified the description in **page 15 line 14**.

Note that equation (1) is a dataset that we would like to predict, not a part of reservoir computing operation. That is, we evaluated whether our FeFET reservoir can predict the time-series dataset explained by equation (1). In actual applications, equation (1) will be replaced by any datasets that you want to predict, such as audio data.

page 15, line 14: In this work, the generation of the voltage waveforms and the conversion to the reservoir states $\mathbf{x}(n)$ were carried out experimentally (see Methods-Electrical measurements), while the calculation of equations (2)-(5) was performed in software as a demonstration.

==

Note on other revised contents

- In our additional experiment for the reviewer-response report, we could improve the measurement precision by reducing the electrical noise during measurement. As results, our overall performance is slightly improved from 14 to 15 classified groups in the t-SNE plot (Fig. 3), C_{STM} from 2.3 to 2.35, C_{XOR} from 2.2 to 2.29, C_{PC} from 2.2 to 2.23 (Fig. 4), NSME of the second-order nonlinear dynamical task from 8.3×10^{-4} to 7.3×10^{-4} (Fig. 5), and other related numbers. There is no change in the main discussion and conclusion of this Article resulted from this improvement.

page 7, line 2: the reservoir states can be separated into approximately 15 groups, indicating that 15 patterns of reservoir states are distinguishable under binary input time-series. We employ the k-nearest neighbor (kNN) method to confirm the 15 distinguishable patterns of the reservoir states in a more quantitative manner.

page 25, line 8: 15 groups of clusters can be observed as indicated by circled alphabets.

page 8, line 7: The short-term memory capacity, defined by[40] $C_{\text{STM}} = \sum_{\tau=1}^{\infty} r^2(\tau)$, is estimated to be 2.35

…

page 8, line 23: The temporal-XOR capacity (C_{XOR}) and the parity-check capacity (C_{PC}), defined by the summations of r^2 in the similar way to the short-term memory capacity[42], are estimated to be 2.29 and 2.23, respectively.

page 10, line 23: The normalized mean squared error (NMSE) for the prediction is estimated to be 7.3×10^{-4},

which is smaller by an order of magnitude than that obtained from a system without the FeFET (see Supplementary Section 10). Note that when using the training dataset as input (same dataset as that for training), the error $y(n) - d(n)$ becomes smaller and NMSE becomes 6.4×10^{-4}.

- The numbering of figure in the previous version of the supplementary might be confusing because it is not related to the section number, for instance Fig. S2, S3, S4 is in the Section 2 and Fig. S5 in the Section 3. We have changed the figure numbers so that they refer to the Section number, for instance Fig. S2.1, S2.2, S2.3, S3.1 instead.

Reviewers' comments:

Reviewer #1 (Remarks to the Author):

I would like to thank the authors for their detailed reply to the concerns I have raised previously.

I think that the manuscript has substantially improved, yet I would like to highlight two points that can improve the impact of this nice work.

1. The authors have extensively replied to my comments on a technical level - which has resulted in modifications of the manuscript starting from the result section. A substantial part of my concerns were related to the general nature regarding novelty, not technical criticism - the technical work is and was good. I would recommend that the authors highlight the novelty of their device using the metrics later mentioned in the manuscript. This would lift the rather general discussion at the beginning to something related to their particular work.

2. The authors mention the problems regarding arrays of FeFETs for RC in terms of area and power consumption. Such considerations are usual in the field, yet they entirely ignore the substantial additional hardware that is required to physically create the temporal multiplexing and demultiplexing. Usually (and I am almost certain that even fundamentally) this substantially exceeds what is saved on the reservoir site. In the introduction the authors cite reference [23] for large FeFET arrays. I would suggest the authors extend their conclusion to a discussion how their results would benefit a RC implementation on such arrays - this would substantially increase the relevance of their work.

Reviewer #2 (Remarks to the Author):

I thank the Authors for their thorough answers. My concerns were fully addressed.

Reply to reviewer comments for Manuscript COMMSENG-22-0001A "Reservoir computing on silicon platform with a ferroelectric field-effect transistor"

Kasidit Toprasertpong

The authors would like to thank the editor and the reviewers for assisting in making this manuscript a more complete and succinct document. In our revision, we took into account the two comments by the reviewer as detailed below. In this report, red texts correspond to the revised/added texts in the manuscript, gray texts correspond to the original texts in the manuscript referred here for reference, and brown texts correspond to the reviewers' comments.

Reviewer #1

I would like to thank the authors for their detailed reply to the concerns I have raised previously.

I think that the manuscript has substantially improved, yet I would like to highlight two points that can improve the impact of this nice work.

1. *The authors have extensively replied to my comments on a technical level - which has resulted in modifications of the manuscript starting from the result section. A substantial part of my concerns were related to the general nature regarding novelty, not technical criticism - the technical work is and was good. I would recommend that the authors highlight the novelty of their device using the metrics later mentioned in the manuscript. This would lift the rather general discussion at the beginning to something related to their particular work.*

We would like to thank the reviewer for the useful comments to help improve our manuscript. We have emphasized the following features of FeFET for reservoir computing in the introduction and the beginning of the result parts to help the readers understand the impact of our work.

- **Utilization of the dynamics of polarization/charge dynamics in reservoir computing**

The dynamics in FeFET include polarization domain nucleation, the polarization-polarization domain interaction in the time domain and space domain, the polarization-charge interaction, the percolation path of current made by spatial distribution of polarization, and so on. We believe that the demonstration of the potential of these dynamics in FeFET for reservoir computing in this work opens a new possibility of physical reservoir computing in the reservoir computing research field.

- **FeFET as multi-terminal CMOS-compatible device**

As FeFET is fundamentally a multi-terminal device, it has a high potential to extract the rich polarization/charge dynamics occurring in both the time domain and space domain inside FeFET mentioned above. Furthermore, the multi-terminal feature allows FeFET to be designed in many configurations, as demonstrated in Fig. 5, indicating its high design flexibility. It also presents a capability to fine-tune the reservoir properties of FeFET through the bias on each terminal, providing an interesting approach of

physical reservoir implementation. These properties are hardly achieved by 2-terminal devices such as conventional memristers, thus are unique characteristics of FeFET.

page 4, line 6: Compared with 2-terminal memristors typically employed to implement CMOS-compatible reservoir computing, FeFETs are fundamentally multi-terminal devices, allowing us to exploit both the temporal and spatial dynamics of polarization and charges for reservoir computing, and notably offer a larger design space to tune the reservoir properties through their multi-terminal structure.

page 5, line 9: The interaction between ferroelectric domains at the vicinity of domain walls … is expected to promote the nonlinearity of polarization dynamics in both the space and time domains. …. As a result, the current flowing from each terminal of a multi-terminal FeFET exhibits the temporal nonlinear dynamics and the spatial distribution through the spatial interaction of polarization and the percolation path formed by the polarization state.

2. *The authors mention the problems regarding arrays of FeFETs for RC in terms of area and power consumption. Such considerations are usual in the field, yet they entirely ignore the substantial additional hardware that is required to physically create the temporal multiplexing and demultiplexing. Usually (and I am almost certain that even fundamentally) this substantially exceeds what is saved on the reservoir site. In the introduction the authors cite reference [23] for large FeFET arrays. I would suggest the authors extend their conclusion to a discussion how their results would benefit a RC implementation on such arrays - this would substantially increase the relevance of their work.*

First, we would like to correct our claim. As we are not discussing about the area in the main manuscript, we assume that the reviewer is mentioning our explanation in our previous rebuttal letter. We mentioned in the previous rebuttal letter that reservoir computing using "a single FeFET" has a clear benefit in area and power consumption as compared to reservoir computing using an active analog circuit (such as circuits with multiple op-amps in 10.1109/TNNLS.2014.2311855), as those active circuits consume substantial area and power to obtain reservoir properties. We did not discuss about the area and power of "a FeFET array" in the previous manuscript.

We would like to make some discussion as suggested by the reviewer. When employing a FeFET array in reservoir computing, the total system consideration including area, power, speed, computing performance, and system design has to be carefully taken into account. This will depend on the nature of tasks and applications we are aiming for. If it is a single-input-single-output task and an array is used to improve the computing performance of a single reservoir (like that shown in Supplementary Section 13 or in other literatures), the benefit of the performance improvement as compared to the increase of total area and power has to be carefully considered, as addressed by the reviewer. On the other hand, for tasks that require the data processing of multiple input sequences, "an array of FeFET reservoirs" enables "parallel processing" of those input sequences for improving training/computational throughput of data processing, as we have

shown an example in recent conference paper (Ref. [46]). We believe that the parallel processing is one of notable benefits of a FeFET array in reservoir computing, as reservoir computing is typically employed in systems that need fast training.

We would like to note that the key point of this manuscript is to demonstrate the potential of FeFET for reservoir computing, while we mentioned about a FeFET array as one potential approach but not as the main proposal of this study. The deep discussion on the array demonstration as well as on Ref. [46] would be out of scope of this manuscript, so we decide to add only a short discussion about the total system requirement in **page 11 line 29** and about the parallel computing in **page 11 line 25**.

page 11, line 19: Such system (FeFET array) is very useful because it allows us to further adjust the short-term memory capacity and the nonlinearity corresponding to computational tasks needed to be solved, as long as it satisfies the system area and power requirements.

page11, line 25: Moreover, an array of multiple FeFETs allows parallel processing of reservoir computing for more efficient training and computational of complicated tasks[46].
